# Sequential Colocalization of ERa, PR, and AR Hormone Receptors Using Confocal Microscopy Enables New Insights into Normal Breast and Prostate Tissue and Cancers

**DOI:** 10.3390/cancers12123591

**Published:** 2020-11-30

**Authors:** Miguel Chenlo, Elvin Aliyev, Joana S. Rodrigues, Paula Vieiro-Balo, Manuel N. Blanco Freire, José Manuel Cameselle-Teijeiro, Clara V. Alvarez

**Affiliations:** 1 Neoplasia & Endocrine Differentiation P0L5, Centro de Investigación en Medicina Moleculary Enfermedades Crónicas (CIMUS), Instituto de Investigación Sanitaria (IDIS), University of Santiago de Compostela (USC), 15782 Santiago de Compostela, Spain; miguelangel.chenlo@usc.es (M.C.); joana.sousa@usc.es (J.S.R.); 2 Department of Pathology, Complejo Hospitalario Universitario de Santiago de Compostela (CHUS), Galician Healthcare Service (SERGAS), Instituto de Investigación Sanitaria de Santiago (IDIS), University of Santiago de Compostela (USC), 15706 Santiago de Compostela, Spain; elvinaliyev1985@gmail.com (E.A.); Paula.Vieiro.Balo@sergas.es (P.V.-B.); 3 Department of Surgery, Complejo Hospitalario Universitario de Santiago de Compostela (CHUS), Galician Healthcare Service (SERGAS), Instituto de Investigación Sanitaria de Santiago (IDIS), University of Santiago de Compostela (USC), 15706 Santiago de Compostela, Spain; Manuel.Narciso.Blanco.Freire@sergas.es

**Keywords:** confocal microscopy, TSA, membrane estrogen receptor alpha

## Abstract

**Simple Summary:**

At present, platforms for multiplex immunohistochemistry (e.g., Opal) identify markers in distinct cell populations within a tissue section using multispectral fluorescence and optic microscopy. However, the optic resolution is not enough to colocalize markers at the subcellular level in the main epithelial or cancer population. We use confocal microscopy in multiplex detection of nuclear hormone receptors since they are an important part of the diagnosis and treatment of breast and prostate cancer. Moreover, we increased the quantitative dynamic range and resolution through increasing the signal/noise ration through reducing autofluorescence and increased longer antibody incubations. *ColNu mIHCF* identified distinct patterns of nuclear receptor colocalization in breast cancers. Furthermore, in prostate cancer all cancer epithelium was positive for ERa at the plasma membrane; and in normal prostate a small ERa+/p63+/AR− basal population suggest stem cell commitment to differentiation. *ColNu mIHCF* could be used for improving diagnosis and treatment in cancer.

**Abstract:**

Multiplex immunohistochemistry (mIHC) use markers staining different cell populations applying widefield optical microscopy. Resolution is low not resolving subcellular co-localization. We sought to colocalize markers at subcellular level with antibodies validated for clinical diagnosis, including the single secondary antibody (combination of anti-rabbit/mouse-antibodies) used for diagnostic IHC with any primary antibody, and confocal microscopy. We explore colocalization in the nucleus (*ColNu*) of nuclear hormone receptors (ERa, PR, and AR) along with the baseline marker p63 in paired samples of breast and prostate tissues. We established *ColNu mIHCF* as a reliable technique easily implemented in a hospital setting. In ERa+ breast cancer, we identified different colocalization patterns (nuclear or cytoplasmatic) with PR and AR on the luminal epithelium. A triple-negative breast-cancer case expressed membrane-only ERa. A PR-only case was double positive PR/p63. In normal prostate, we identified an ERa+/p63+/AR-negative distinct population. All prostate cancer cases characteristically expressed ERa on the apical membrane of the AR+ epithelium. We confirmed this using ERa IHC and needle-core biopsies. *ColNu mIHCF* is feasible and already revealed a new marker for prostate cancer and identified sub-patterns in breast cancer. It could be useful for pathology as well as for functional studies in normal prostate and breast tissues.

## 1. Introduction

Multiplex immunohistochemistry has been developed as a complementary diagnostic method in pathology also in its chromogenic or fluorescent-based (mIHCF) forms [1,2,3,4,5,6,7]. mIHCF is based on the HRP-mediated covalent union of TSA to tyrosines [8]. Covalently linked TSA resists alkaline pH or microwave-induced heat; instead, both methods are capable of breaking weak ionic bonds, washing out the antibodies [2,9]. Fluorophore-bound tyramide signal amplification (TSA) molecules are already being used as a substrate for mIHCF in automated commercial systems for pathology diagnostics, after adapting the multispectral fluorescence image acquisition characteristic of confocal microscopy to widefield optical microscopy and the application of quantitative algorithms including deep learning [10,11]. mIHCF has become an essential tool to evaluate the tumor microenvironment, quantify distinct immune populations and predict response to immunotherapy [12,13,14]. This has immediately helped in the prognostic stratification of many cancers either in survival or response to treatment, as for example in breast, lung, gastric, and pancreatic cancer, Hodgkin lymphoma, and angiosarcoma [15,16,17,18,19,20]. However, the methods of automated widefield microscopy currently available use combinations of antibodies that stain different targeted cellular populations within a tissue section by scanning with a widefield optical fluorescent microscope. Instead, we contemplated the possibility of mIHCF testing for colocalization within the same cell using confocal microscopy that could be suitable for pathology diagnostics if antibodies approved by regulatory agencies for clinical in vitro diagnostics (IVD) are employed.

Moreover, if possible, the most difficult combination would be markers that not only stain the same cells, but also the same organelle within those cells. A previous publication had performed triple mIHCF using confocal microscopy, although only two of the markers were co-expressed in one cell and none in the same organelle [21].

Nuclear markers are commonly used as tools in diagnostics. Sex hormone nuclear receptors, estrogen receptor α (ERa), progesterone receptor (PR), and androgen receptor (AR) are essential biomarkers in breast and prostate cancer pathology. These markers are indicators and/or targets of effective therapies in various clinical settings, making an accurate, essential assessment for patient management [22,23,24]. Some previous works have colocalized ERa and PR in breast cancer or ERa and AR with other markers in colon cancer using optical microscopy and multispectral detection using non-IVD primary and different species-specific secondary antibodies [1,2].

However, we endeavored to detect three nuclear receptor or other nuclear markers in the same tissue section of breast and prostate using routine IVD reagents for pathology diagnoses, i.e., diagnostic primary antibodies with a common secondary antibody preparation for both mouse and rabbit sources. Nuclear receptors could be nuclear, cytoplasmic, or both, and confocal microscopy is required for subcellular colocalization. Moreover, using this methodological approach, there is a distinct possibility that the same cell population can be labelled by all three markers.

The focus of our study was to design and standardize a technique to colocalize nuclear hormone receptors and other transcription factors in the same tissue section using validated IVD antibodies in multiplex staining. Once standardized, we wanted to explore its usefulness in the study of normal breast and prostate tissues and cancers.

## 2. Results

### 2.1. ColNu mIHCF Standardization

Autofluorescence of human FFPE sections is a technical problem when dealing with a highly sensitive system, such as immunofluorescence. Previous works in the literature address this problem through incubation in different solutions that quench or cover this background fluorescence, such as CuSO4 or Sudan Black B (SBB), respectively [25,26,27,28]. Some authors use SBB prior to immunofluorescence [28], while other do so at the end of the procedure, just before mounting, to prevent interferences with antibody binding [25,26,27]. We tested these methods in an initial tissue micro-array 0 (TMA-0) containing normal thyroid tissue with high background due to the intrafollicular colloid using our previous protocols [29,30,31,32,33]. As shown in Figure S1 the best method for having the highest signal/noise ratio was 0.1% SBB.

We sought to test three cycles of staining/detach antibodies for nuclear receptors in a hospital setting, that would be practicable in routine clinical practice. Thus, the technique should follow protocols that did not exceed conventional working times and rely on validated commercial hormone receptor antibodies for diagnostic IHC with DAB. These commercial ready-to-use preparations rely in very tight dilutions, and short incubation times (<1 h) to detect nuclear hormone receptor abundance for selecting cancer cases known to respond to anti-hormonal therapy. These precise dichotomous (yes/no) conditions confer high specificity, impeding false positives in optical chromogenic microscopy. However, confocal microscopy detects an ample range of fluorescent intensities, thereby enabling quantitative detection. Therefore, we aimed to detect the entire receptor expression in both normal (low expression) and cancerous tissue (from negative to high expression). For both of these reasons (cycle length; sensibility), our IVD primary antibodies were incubated overnight in all three consecutive cycles.

Several blocking solutions were assayed that could improve specificity by detecting low levels of ERa and PR in normal breast tissue in conventional IHC with overnight incubation of the primary antibody. We compared the obligatory commercial peroxidase/blocking solution alone or followed by 0.1% BSA or 0.1% I-block (purified casein) for two hours. All three yielded adequate results in regular IHC, albeit the I-block appear to yield a completely clean stroma without remnants of DAB. We therefore added this blocking step to our protocol.

The next step to standardize mIHCF using fluorescent TSA, was to wash out the antibodies after the first cycle and test if the remaining signal was strong enough for confocal microscopy. We adapted the protocol of the group of Mezey combining high pH with microwave heating [9]. The variables we found to be important for homogeneous washing out of the antibodies while maintaining strong TSA signal and tissue section well adhered to the glass slide were: (a) time inside the microwave, (b) the size and type of box used, (c) the volume of buffer in the box, and (d) number of glass slides treated together inside the box. Thenceforth, the time inside the microwave was controlled with a lab timer; buffer volume was precisely measured, and the number of glass slides inside the box was kept at four with empty slides replaced when needed. To prevent cross-contamination with wash-out antibodies in different slides, negative controls omitting the first antibody in any of the three cycles or washing-out of different primary antibodies, were performed in different boxes.

**Figure 1:**
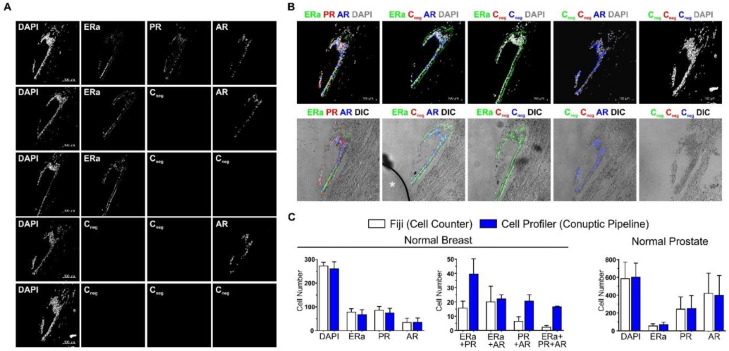
*ColNu mIHCF* for nuclear hormone receptors with negative controls for each cycle. (**A**) Confocal maximal projections after sequential staining for ERa, PR, and AR in normal breast tissue performed on one slide while omitting one or all primary antibodies (Cneg) in parallel slides. (**B**) Overlay of the channels with DAPI (top) or phase contrast (DIC, bottom). * white asterisk indicates air bubble in the mounting medium. Scale bar shown in DAPI channel. (**C**) Quantification using Fiji or a designed pipeline with Cell Profiler. No differences were found in single channels in normal breast or prostate. Cell profiler was more sensitive for double or triple colocalization. The order of antibodies in the consecutive cycles is indicated above each photograph. Pseudocolor was kept constant: green for the 1st, red for the 2nd, and blue for the 3rd cycle. DAPI was included in the mounting medium and reveals the nuclei. DIC is a plane obtained with transillumination for differential interfering contrast-Nomarski and reveals tissue structure.

Having established the protocol, we performed *ColNu mIHCF* in two tissue microarrays (TMAs), TMA-I and TMA-II keeping to a systematic order: first ERa (expected strong signal in breast, no signal in prostate) followed by PR (intermediate signal in breast expected), and, finally, AR (strong signal expected in prostate; lower signal in breast) (Figure 1). However, we also performed alternative arrangements to include other nuclear markers, such as TTF1 or p63. DAPI was included with the mounting medium for labelling nuclei. *ColNu mIHCF* performed in parallel slides with all three cycles were compared to substituting 1^st^ antibody for diluent in either of the cycles (Cneg). Figure 1A shows the signal recorded for ERa, PR and AR in normal breast tissue at their corresponding wavelength channel. Omitting primary PR antibody in the second cycle prevents any signal in this channel, but does not affect AR staining during the third cycle. Omitting primary PR and AR antibodies in the second and third cycles block any signal in the two corresponding channels. Omitting primary ER and PR antibodies in the first and second cycles gives no signal for those antibodies, while leaving the signal of AR from the third cycle unaffected. Finally, as expected for an IVD immune reagent, the secondary antibody *per se* does not yield an unspecific signal even after repetitive cycles of staining. We assigned a pseudocolor to each channel and overlaid them with DAPI or with a phase contrast plane (differential Interfering contrast-Nomarski, DIC) to reveal tissue structure (Figure 1B). Colocalization of two or more nuclear receptors is seen in the epithelial cells. Stromal cells reveal less intense staining. We applied systematic quantification to the five normal breast and prostate *ColNu mIHCF* using the Fiji *Cell Counter* tool, or with a pipeline of Cell Profiler (Figure 1C). No differences were observed between the two systems per single channel, although automatic counting was more sensitive for double/triple colocalization. With these tools, epithelial and stromal tissue components in each picture could be counted separately (Figure S2). Detailed qualitative descriptions of our findings for paired cases of normal breast and breast cancer and normal prostate and prostate can be found below.

### 2.2. ColNu mIHCF in Normal Breast (NB) and Breast Cancer (BC)

**Figure 2:**
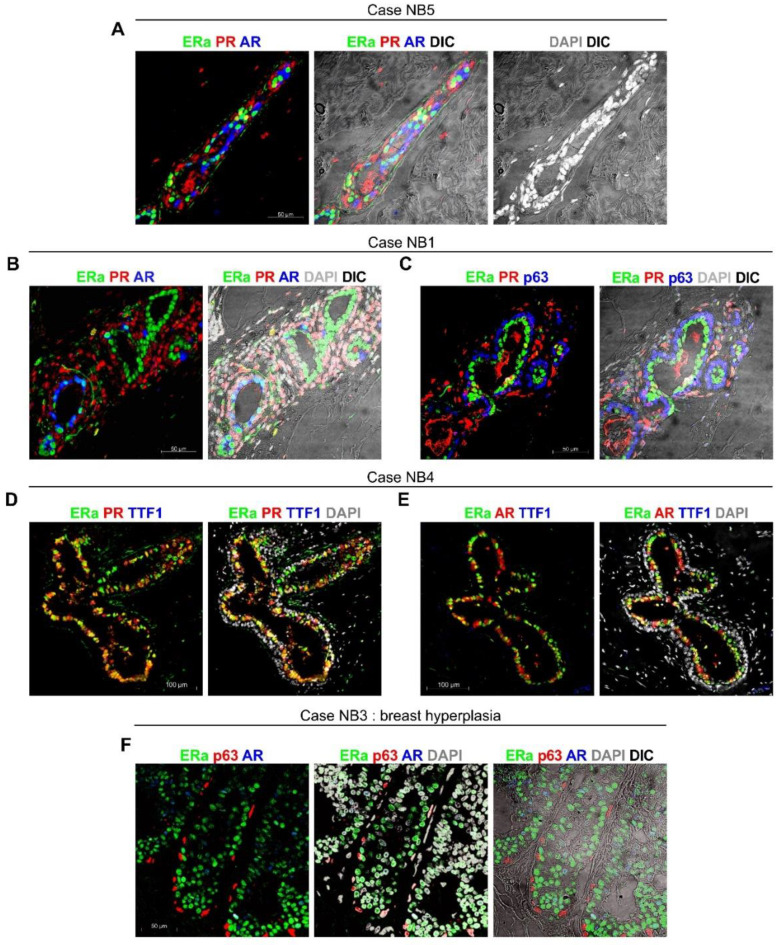
*ColNu mIHCF* in normal breast tissue from paired cancer cases. (**A**) NB5 reveals some epithelial cells co-expressing ERa + PR; others co-express ERa + AR, while many are single positive for ERa, PR, or AR. Stroma was positive for PR. (**B**) NB1 shows many epithelial cells single positive for ERa, with some colocalizing ERa + AR. PR+ cells accumulated in the stroma, suggesting an early alteration. The tissue maintained the basal p63 layer (**C**) showing some positivity for PR. (**D**,**E**) NB4 also presents many cells co-expressing ERa + PR (**D**) and others co-expressing ERa + AR (**E**), while the basal layer is negative for hormone receptors. TTF1 is negative in the epithelium and inconsistently positive in the stroma. (**F**) NB3 shows hyperplasia of the ERa epithelium with some population co-expressing ERa + AR. The basal p63 layer, negative for hormone receptors, is maintained. The order of antibodies in the consecutive cycles is indicated above each photograph. Pseudocolor was kept constant: green for the 1st, red for the 2nd, and blue for the 3rd cycle. DAPI was included in the mounting medium and reveals the nuclei. DIC is a plane obtained with transillumination for differential interfering contrast-Nomarski and reveals tissue structure.

*ColNu mIHCF* for ERa, PR, AR, p63, and TTF1 combined in groups of three markers were performed in TMA-I and TMA-II arrays (Table S1). In normal breast tissues, a constant population of epithelial cells were found to co-express ERa + AR (Figure 2A,B,E). As for the co-expression of ERa and PR, some epithelium presented high colocalization (Figure 2D), while others low (Figure 2B). p63 was not co-expressed with ERa or AR (Figure 2C,F), but slightly with PR in case NB1 (Figure 2C). TTF1 positivity was practically non-existent in normal breast, except for a few isolated cells with low signal (Figure 2E). Case NB1 exhibited a population of PR-positive stromal cells surrounding the basal p63+ layer, suggesting an activated state that differs from true normal breast tissue (Figure 2C). Case NB3 was considered to be breast hyperplasia, with few cells co-expressing ERa + AR (Figure 2F).

**Figure 3:**
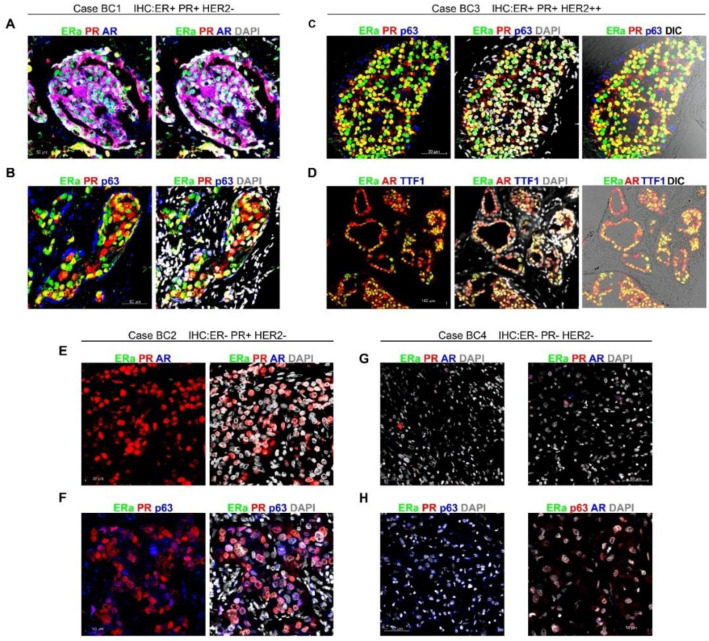
*ColNu mIHCF* in breast cancer from different hormone receptor and HER2 status. (**A**,**B**) BC1 presents ductal carcinoma in situ (DCIS) area maintaining the basal p63 layer. Epithelium co-expresses nuclear ERa with strong whole-cell (cytoplasmic + nucleus) PR and AR localization. In A (top-left), the blended white color indicates overlay in quantity and location of all three receptors. (**C**,**D**) BC3 is another DCIS area with an intact p63 basal layer. All three receptors, ERa + PR + AR, are co-expressed exclusively in the nucleus, as shown by the blended yellow color in ER + PR (**B**) and in ER + AR (**D**). TTF1 was negative, as were all the other breast cancer cases. (**E**,**F**) BC2 epithelium (invasive ductal carcinoma) expresses PR exclusively, being negative for both ERa and AR. The PR+ population presents a p63+ sub-population. (**G**,**H**) BC4, a triple negative invasive ductal carcinoma, shows negativity for all three nuclear receptors. The cancer epithelium expresses p63. Shown are two independent fields for each multiple combination of antibodies. The order of antibodies in the consecutive cycles is indicated above each photograph. Pseudocolor was kept constant: green for the 1st, red for the 2nd, and blue for the 3rd cycle. DAPI was included in the mounting medium and reveals the nuclei. DIC is a plane obtained with transillumination for differential interfering contrast-Nomarski and reveals tissue structure.

*ColNu mIHCF* in breast cancer coincided mostly with the diagnostic IHC in terms of nuclear receptor expression; however, we attained new qualitative information related to colocalization and subcellular distribution (Table S1 and Figure S3 for individual signal of each channel). BC1 and BC3 correspond to areas of ductal carcinoma in situ (DCIS) from two cases of invasive ductal carcinomas of no special type (NST). BC1 and BC3 were diagnosed as DCIS ERa+/PR+ with HER2 negative in BC1 but positive in BC3 (Table S1). *ColNu mIHCF* revealed that both cases presented high co-expression of ERa with PR and AR (Figure 3A–D and Figure S3). The basal p63 layer was negative for hormone receptors in both cases (Figure 3B,C).

There was a difference between the two cases, however. BC3 had strong nuclear-exclusive colocalization of the three receptors (Figure 3C,D), while BC1 presented nuclear ERa and strong whole cell (cytoplasmic + nucleus) PR + AR co-expression (Figure 3A,B). In the three channel overlay, many cells in BC1 presented white nuclei (ERa + PR + AR) with purple cytoplasm (PR + AR). The two cases had markedly different Ki67 index – BC1 = 40% while BC3 = 15% (Table S1).

BC2 and BC4 were invasive ductal breast carcinoma of NST. BC2 was only PR+ and negative for any other nuclear hormone receptors (Figure 3E), but exhibited one epithelial population co-expressing PR and p63, while a second population was PR+ only (Figure 3F). BC2 Ki67 index was 37% (Table S1).

BC4 had been diagnosed as triple negative case with Ki67 index of 71% (Table S1). In *ColNu mIHCF* nuclear hormone receptors were consistently negative, but the entire epithelial population was p63+ (Figure 3G,H).

**Figure 4:**
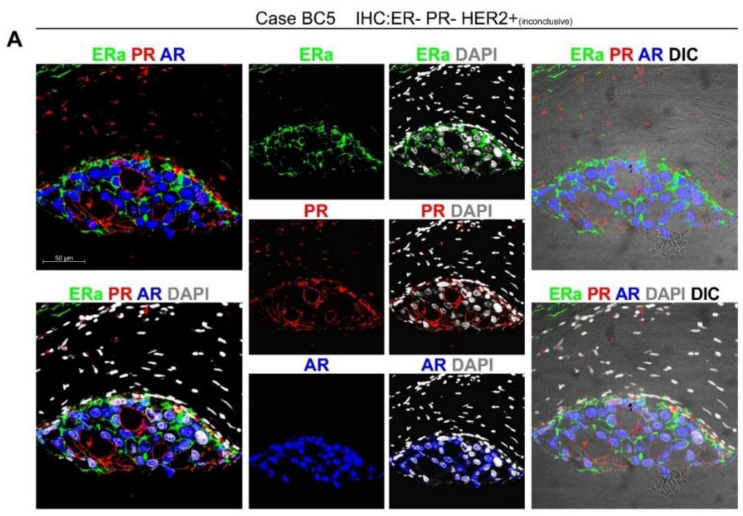

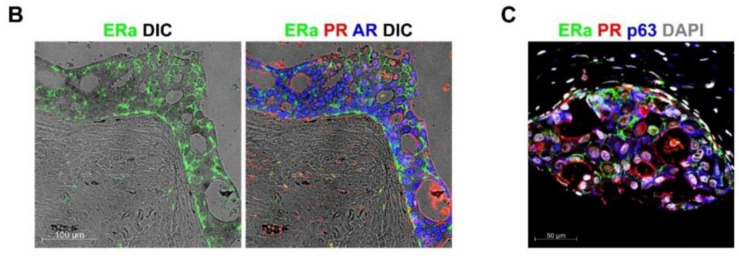
A breast cancer case diagnosed as triple negative presents plasma membrane ERa expression in *ColNu mIHCF*. BC5 is a case of ERa/PR-negative invasive ductal carcinoma with low HER2 positivity, and negative FISH. (**A**,**B**) In DCIS areas, *ColNu mIHCF* reveals epithelium with ERa located on the plasma membrane, strong nuclear AR with some PR colocalization, and PR also located on membrane regions not in contact with other epithelial cells. A basal layer of cells in contact with the stroma was strongly PR+. (**C**) Tumor cells also shows express p63. At the stroma, the most abundant population is PR+ at a distance from the cancer (**A**,**B**) and p63 in the vicinity (**C**). The order of antibodies in the consecutive cycles is indicated above each photograph. Pseudocolor was kept constant: green for the 1st, red for the 2nd, and blue for the 3rd cycle. DAPI was included in the mounting medium and reveals the nuclei. DIC is a plane obtained with transillumination for differential interfering contrast-Nomarski and reveals tissue structure.

BC5 illustrated areas of DCIS within an invasive ductal breast carcinoma of NST and was diagnosed as negative for ERa/PR and classified as triple negative, after a dubious HER2 (weak positivity) IHC, and negativity for HER2 FISH (Table S1). *ColNu mIHCF* for nuclear hormone receptors detected plasma membrane localization of ERa in the epithelium that also presented strong nuclear AR (Figure 4A,B). There were fewer cells expressing PR located at the plasma membrane of those cells not in contact with other cells (Figure 4A). There was a basal layer of cells expressing PR (Figure 4A,B). The entire cancer epithelium was positive for p63 (Figure 4C). At the stroma, the most abundant population was PR+ and in the vicinity of the cancer, there was p63 positivity (Figure 4A–C).

The striking ERa staining (and PR) at the plasma membrane in one (BC5) of the two triple negative cases (BC4, BC5), led to the interesting question as to whether it would be a frequent pattern in triple negative breast carcinomas. To explore this possibility, we studied a new array with four other triple negative cases (TMA-III cases BC6-BC9), three from breast and one lymph node metastasis (Figure S4, Table S1). All four cases were positive for AR with more or less intensity, but only one case (BC7) co-expressed weak nuclear ERa but not at the plasma membrane. In the medullary carcinoma case (BC8) and the metastatic case (BC9), an AR + PR double positive population appeared. These pleomorphic cancer cells presented intense cytoplasmic positivity for PR with the nucleus excluded, and had a dendritic-like appearance. These results suggest that *ColNu mIHCF* could contribute to the sub-stratification of triple negative breast cancers in future studies.

### 2.3. ColNu mIHCF in Normal Prostate (NP) from Paired Cancer Cases: ERa Marks a p63 + Basal Cell Subpopulation

**Figure 5:**
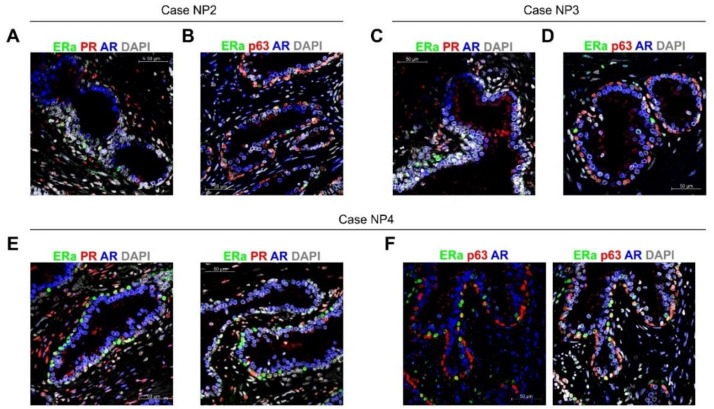

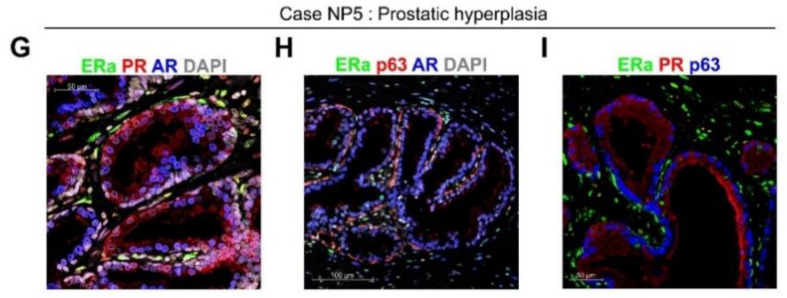
*ColNu mIHCF* in normal (non-malignant) prostatic tissue from paired cancer cases identifies a basal cell co-expressing p63 and ERa. (**A**) NP2 illustrates the prostate epithelium expressing AR with a few isolated ERa+/AR− epithelial cells. PR is expressed at the stroma together with some ERa positive cells. (**B**) ERa epithelial cells are basal cells co-expressing p63 as seen by the blended yellow color. Few of these cells express greater amounts of ERa than p63, resulting in bright green. The basal layer is p63+, but AR-. The same pattern is repeated in cases NP3 (**C**,**D**) and NP4 (**E**,**F**). (**G**) NP5 illustrates a different staining pattern in which the AR+ epithelial cells co-express PR. In addition to the few basal ERa+ cells, a layer of ERa+ elongated cells is also observed. This sample was considered prostate hyperplasia. (**H**) The two types of ERa+ cells are shown at lower magnification. Isolated basal epithelial cells co-expressing p63 and ERa and another layer of elongated ERa in the proximity of the basal layer. (**I**) At high magnification, the prostate epithelium expressing PR, the p63 layer with discrete ERa+ cells, and the second layer of ERa elongated cells p63− can be seen. The order of antibodies in the consecutive cycles is indicated above each photograph. Pseudocolor was kept constant: green for the 1st, red for the 2nd, and blue for the 3rd cycle. DAPI was included in the mounting medium and reveals the nuclei).

*ColNu mIHCF* was performed in NP from TMA-I and TMA-II arrays (Table S2). Cases NP1-NP4 showed a strong nuclear signal for AR in the epithelium with discrete basal cells with intense nuclear positivity for ERa (Figure 5A–F). These ERa+ cells at the basal layer co-expressed p63 at low intensity (Figure 5B,D,F). PR was poorly expressed in some AR epithelial cells but was abundant in the stroma.

Case NP5 exhibited different histology and staining. It was diagnosed to be benign prostate hyperplasia (Table S2). *ColNu mIHCF* revealed that the AR epithelium co-expressed abundant PR (Figure 5G,I). Moreover, in addition to the few separate double-positive ERa/p63 basal cells, there was an extensive p63- ERa+ population, under the basal layer (Figure 5G–I).

### 2.4. ColNu mIHCF in and Prostate Cancer (PC): Apical ERa is a Cancer Marker

**Figure 6:**
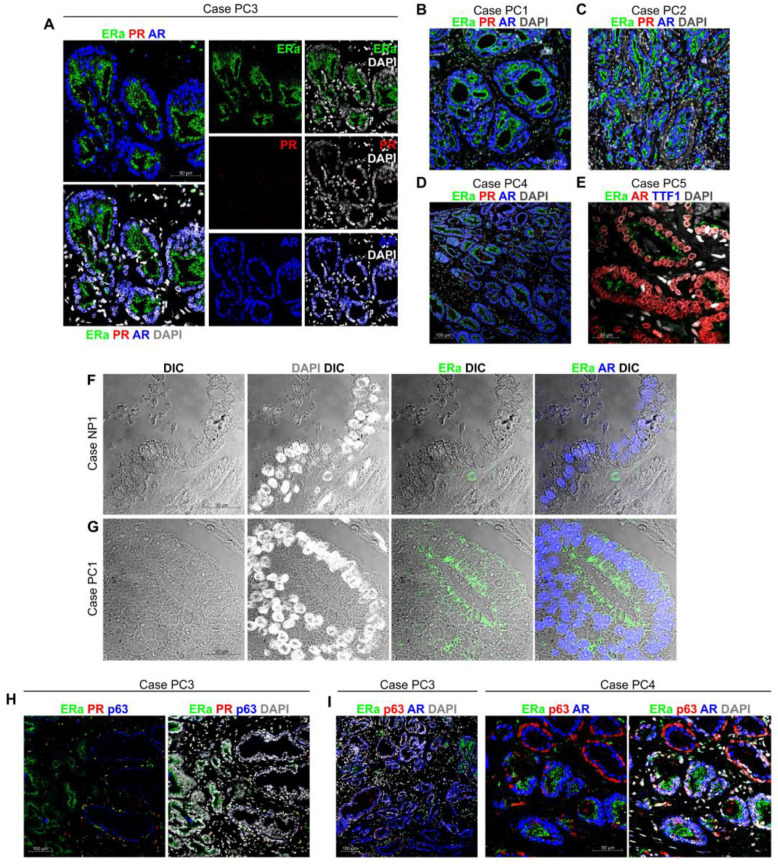
*ColNu mIHCF* in prostate cancer reveals ERa at the apical membrane of cancer epithelium. (**A**) *ColNu mIHCF* for all three nuclear receptors in PC1 showing each separate channel (center) and the overlays among them (left) or with DAPI (right). Prostate cancer epithelium expresses AR (blue) in the nucleus, but ERa (green) on the apical membrane. PR is not expressed. (**B**–**D**) Lower magnifications reveal the same pattern of nuclear AR and apical membrane ERa co-expression in PC1 (**B**), PC2 (**C**), and PC4 (**D**). (**E**) Similar staining with different order and TSA for AR, again exhibits nuclear AR (red) and apical membrane ERa (green) co-expression in the cancer epithelium of case PC5. TTF1 staining was negative. (**F**,**G**) A phase contrast plane (DIC) is shown for paired samples of case NP1/PC1 alone or overlaid with other channels. A single basal ERa cell is shown underneath the normal prostate epithelium expressing nuclear AR in NP1 (**F**). The whole prostate cancer epithelium co-expressing nuclear AR and apical membrane ERa is shown in PC1 (**G**). (**H**) At lower magnifications (case PC3), the combination of p63 and ERa demarcate prostate cancer from normal prostate tissue. On the left, prostate cancer with ERa located on the apical membrane. On the right, normal prostate epithelium with the p63 basal layer displaying some p63/ERa double positive cells. PR is expressed at the stroma. (**I**) The same result was obtained for PC3 and PC4. While AR is located in the nucleus of both cancer and normal prostate epithelial cells, apical membrane ERa staining is specific for cancer, while p63 is conserved at the intact basal layer of normal prostate tissue. The order of antibodies in the consecutive cycles is indicated above each photograph. Pseudocolor was kept constant: green for the 1st, red for the 2nd, and blue for the 3rd cycle. DAPI was included in the mounting medium and reveals the nuclei. DIC is a plane obtained with transillumination for differential interfering contrast-Nomarski and reveals tissue structure).

For prostate cancer, cases with different Gleason score were included in our study since they correspond to progressive loss of differentiation that are related to particular molecular expression patterns [34]. All prostate cancer cases studied with *ColNu mIHCF* presented homogenous staining (Figure 6 and Figure S5 for each channel-specific signal). ERa was exhibited at the plasma membrane of the apical pole in prostate cancer epithelium co-expressing nuclear AR (Figure 6A). PR was negative at the epithelium and stained a few stromal cells. This particular staining was repeated in all cases tested (Figure 6B–E), while TTF1 was negative.

Figure 6F,G presents paired samples from normal prostate (NP1, Figure 6F) and prostate cancer (PC1, Figure 6G) displaying ERa, AR or DAPI channels combined to DIC. The normal epithelium expressed nuclear AR but no ERa. At the basal layer negative for AR, a single cell positive for nuclear ERa (Figure 6F). The prostate cancer epithelium also express nuclear AR but co-expressed ERa at the apical pole in each cell (Figure 6G).

Systematic observation of ERa/PR/AR *ColNu mIHCF* of prostate cancer with lower magnification (20×) enabled certain areas where nuclear AR was present in the absence of ERa to be distinguished. Normal prostate tissue appeared to be interspersed in cancer tissue. To confirm this, we performed *ColNu mIHCF* including p63 to reveal the basal cell layer still present in normal prostate epithelium but absent in prostate cancer (Figure 6H,I). ERa/PR/p63 *ColNu mIHCF* discriminated precisely the epithelium with ERa at the apical pole (cancer) from the epithelium with a layer of p63 basal cells, some of which were positive for nuclear ERa (Figure 6H). PR was expressed by scattered stromal cells. ERa/p63/AR *ColNu mIHCF* revealed that ERa was as clear a marker of prostate cancer as p63 is for normal prostate epithelium (Figure 6I).

### 2.5. Conventional IHC of ERa in Prostate Cancer in Tissue Sections and Needle-Core Biopsy

**Figure 7:**
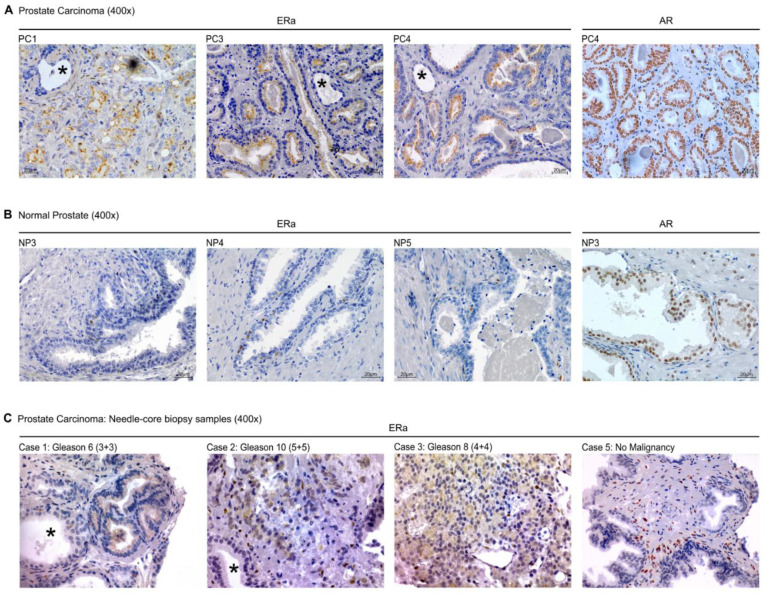
Immunohistochemistry with *ColNu mIHCF* conditions confirms ERa as a marker of prostate cancer. (**A**) Representative microphotographs of prostate cancers from TMA-I and II performed with overnight incubation of the primary antibody (*ColNu mIHCF* conditions). Left: Cancer epithelium is positive for ERa at the apical membrane, while normal prostate epithelium is not. Right: Nuclei of cancer and normal epithelial cells are positive for AR. (**B**) Left: Normal prostate epithelium is negative for ERa. At the basal layer, isolated cells show positivity for ERa. Right: Prostate epithelial cells in normal tissue express nuclear AR. (**C**) Sections from prostate needle-core biopsies similarly stained for ERa showing the stained prostate cancer while the normal tissue (asterisk) is negative or presents isolated nuclear ERa positive cells at the basal layer. Right: a case with no malignancy exhibits a proliferation of ERa cells within the stroma. (*, asterisks indicate normal prostate epithelium).

We followed up on the idea that the particular apical staining of ERa could be a useful marker for prostate cancer. Consequently, we performed IHC with DAB for ERa with similar conditions to the ones used for *ColNu mIHCF* (overnight incubation of the Ready-to-Use IVD antibody) in all the prostate cancer samples from TMA-0, TMA-I and TMA-II arrays (globally, nine cases of prostate cancer). All cancer samples exhibited apical ERa staining (Figure 7A) that was not present in normal prostate, where the odd basal ERa+ cell was also detected (Figure 7B). ERa apical staining easily distinguished cancer from normal epithelium in the same field, a feature that was impossible with IHC for AR (Figure 7A,B, asterisks).

We compared ERa IHC performed with the blocking step as *ColNu mIHCF* (commercial peroxidase/blocking solution followed by 0.1% I-Block) against commercial peroxidase/blocking solution alone without any other additional blocking step. The result was identical, indicating that the key factor was overnight incubation of the ERa antibody. Thus, this staining could be incorporated immediately into routine clinical practice.

Next, we performed ERa IHC (overnight incubation) in sections from needle-core biopsy (Table S3). Of six cases, the four where prostate cancer was seen (Gleason: 6–10) exhibited apical ERa (Figure 7C), while the two cases not showing cancer did not present apical ERa. Case 5, diagnosed as “no malignancy”, displayed abundant stromal ERa positivity reminiscent of the previously described case of prostatic hyperplasia in case NP5 sections (see Figure 5I).

## 3. Discussion

We have established a multiple IHC technique for nuclear hormone receptors and other nuclear markers that may be colocalized within the same cell. *ColNu mIHCF* uses long primary antibody incubations, fluorescence detection and confocal microscopy, resulting in a highly quantitative technique.

Most of our standardization has been done with three consecutive staining cycles; albeit we know that a fourth cycle is possible, as we have tested it with cytokeratin staining (AECK or AE1-AE3). We have combined the detection of three nuclear hormone receptors (ERa, PR and AR), a thyroid transcription factor (TTF1, which must be negative in prostate and breast, but positive in thyroid tissue), and p63 (the characteristic basal cell transcription factor). Each positive or negative control yielded the expected result in all of the cycles tested. This implies that the colocalization results obtained for nuclear hormone receptors are reliable.

The two breast cancer cases classified as ERa+ (BC1 and BC3) co-expressed abundant PR and AR in the same cell. This did not occur in normal breast tissue where the luminal epithelium frequently co-expressed ERa + AR or, less frequently, ERa + PR, but rarely all three receptors. Furthermore, we found a difference between both ERa positive cases in the sub-cellular localization of the PR and AR receptors. While in BC3 all three receptors were concentrated within the nucleus, in BC1 PR and AR were found throughout the whole cell (cytoplasm and nucleus). Intriguingly, BC1 has more than twice the Ki-67 index than BC3, which was also positive for HER2. While we do not know if any or both of these differences are related to the nuclear/cytoplasmic pattern of receptor colocalization in DCIS, it does deserve to be investigated in greater depth with large series of cases. At the molecular level, crosstalk among the three nuclear receptors has been reported and hormone receptors are capable of binding to common co-activators/corepressors [35]. Additionally, new compounds acting either as ERa modulators or degraders, AR modulators or antagonists, and PR modulators are under clinical trial for treatment-resistant breast cancer or to prevent progression [36]. Perhaps a good characterization of co-expression and subcellular location in the initial sample in relation to follow-up and recurrence would provide insights into mechanisms of resistance and/or aid in the selection of the appropriate therapeutic avenue.

PR has a controversial role in breast tissue expressing ERa [35]. In normal breast tissue, progesterone reduces estrogen-induced proliferation through endocrine and paracrine interactions [37,38]. Many data in cellular and animal breast cancer models suggest that progesterone exert a cancer promoter role by acting on MYC and CCDN1 genes [39]. The number of PR+ cells in a normal breast biopsy correlates significantly with the breast cancer incidence nine years later [40]. However, in ERa+ breast cancers, PR is a predictive marker of better treatment response, although exclusively in premenopausal women [35,41]. It has been posited that unbound PR contributes to the ERa proliferative role, while ligand-bound PR would inhibit ERa cancer promotion. Thus, it could well be that both PR antagonists and agonists or modulators have a role on the treatment-resistant ERa+ breast cancer in postmenopausal patients [35,36]. Nonetheless, of the two isoforms, PR-B and PR-A –a similar protein lacking the 164 initial aminoacids- PR-A is known to be the most associated to protumoral collaboration with ERa, and a Phase II clinical trial with PR modulators is underway for PR-A overexpressing breast cancers [36]. There are no studies regarding PR localization within the cytoplasmic. Given that our PR antibody recognizes both isoforms, the cytoplasmic localization could be a result of the predominance of either isoform.

AR also has a controversial role in ERa+ breast cancer. In a normal breast, AR counteracts the proliferative action of ERa [42]. Furthermore, AR is an independent predictor of survival for ERa+ breast cancers sufferers [43]. In contrast, AR is involved in tamoxifen resistance in ER+ breast cancers and AR inhibitors are under clinical trial [44,45]. Subcellular localization has yet to be factored in, but we have recorded differences between case BC3, displaying the co-existence of nuclear AR with ERa and PR, and case BC1, where AR was in the cytoplasm together with PR while colocalizing with ERa + PR in the nucleus.

Case BC2 case expressed PR in the absence of any other nuclear receptor. This is a rare group of breast cancers that is not well studied [41,46,47]. There were two PR populations co-existing in this case: a p63+ one and another p63−. To the best of our knowledge, this is the first report of these patterns of co-existence that will require further study. Between 10–15% of high-grade, triple negative basal breast carcinomas with metaplasia express low levels of p63 as in our BC4 case [48].

The last breast cancer case, BC5, expressed ERa, although it was located on the membrane. This case had been diagnosed as triple negative. ERa isoforms 3 (ERa-36) and 4 (ERa-46) are shorter isoforms lacking the N-terminal half (1-173 a.a.) and are able to be expressed and signal on the plasma membrane [49]. Nevertheless, the epitope of our anti-ERa antibody is located in the N-terminal portion, exactly between a.a.37–42 [50] recognizing full-length isoform 1 (ER-66) and isoform 2 (lacking C-terminal, low expression in general). This case had some HER2 expression classified as inconclusive. Non-genomic effects of steroids are known to be mediated by membrane-associated or cytoplasmic ERa, PR or AR in many types of cells, including breast cancer, activating kinase pathways such as ERK or PI3K/AKT (reviewed in [51,52]. Moreover, it has been shown that the three hormone receptors (ERa, PR and AR) are able to interact with each other in the cytoplasm of different breast cancer cells to control many biological responses induced by sex steroids [52,53]. ERa can associate to HER2 to promote vascular growth factor receptor ligands [54,55]. Importantly, when there is loss of nuclear co-expression of ERa and AR receptors, as in the triple negative case BC5, AR expression is considered a factor of poor prognosis and aggressiveness [43] able to induce invasiveness in in vitro models [56]. Of notice, of the triple negative cases studied with *ColNu mIHC* in this study (BC4, BC5 and the four cases in array TMA-III) five out of six were positive for AR. Only two presented strong plasma membrane (BC4) or weak nuclear (TMA-III-BC7) ERa expression. Again, this is merely a descriptive result of a small group of cases and more studies are needed with larger series of triple negative breast cancers.

Our results for the prostate have been consistent. The cells of the normal (not malignant) prostatic glands have an ERa+ nuclear subset among its basal population. These cells have previously been described as a small population (<10%) among basal cells [57,58]. We have enriched this description further by adding that it is an ERa+/p63+_low_/AR− population. In mice, injection of high doses of estradiol or testosterone induces prostate cancer; however, this does not happen in knockout mice for ERa, nor in aromatase knockouts in which hyperplasia and hypertrophy are observed, but not cancer [59]. Interestingly, injection of estradiol produces basal cell metaplasia [60]. These results do not incontestably answer the question of whether the fundamental receptor in the normal prostate is ERa or ERb. Recently, in vitro culture of stem cell spheres from normal human prostate tissue has shown that they express ERa [61]. This suggests that *ColNu mIHCF* staining identifies prostate stem cells on which it would now be possible to conduct numerous functional and quantitative studies. Case NP5 case exhibited areas of hyperplasia with abundant ERa+ cells at the stroma, also reported in a recent study [58]. In old rats, positivity for aromatase and ERa correlates with the presence of hyperplasia and prostatitis, characteristic of age [62]. More studies are needed to probe into the ERa cell populations in normal or benign prostate hyperplasia.

All prostate cancer samples displayed ERa expression on the apical pole of AR+ epithelium. We have replicated this result numerous times, with different samples from several TMAs, with two different techniques (*ColNu mIHCF* and IHC) as well as in other types of prostate samples such as needle-core biopsies and full-length sections of surgical specimens. We believe that this labeling is quite simple to implement in routine prostate cancer assessments, since the anti-ERa antibody is commonly found in clinical pathology laboratories. The only requirement is longer incubation times with ERa primary antibody. ERa staining accurately discriminates cancerous tissue from normal tissue and can be invaluable in needle-core biopsies, as well as in delimiting cancer margins in surgical specimens more easily.

Several earlier works have studied ERa expression in prostate cancer tissues [58,63,64]; however, the data always refer to nuclear ERa using IHC while the presence of membrane ERa is not addressed. Non-genomic effects of estradiol have also been described in cellular models of prostate cancer where they promote epithelial-mesenchymal transition [59,65].

We discovered this membrane ERa receptor thanks to the use of confocal microscopy, which enables precise viewing of different subcellular regions. Now, having discovered the conditions necessary, identifying membrane ERa receptors within the apical pole of the prostate cancer cell in conventional widefield microscopy has been simplified. This staining can be immediately implemented in clinical pathology laboratories, although its true usefulness will be determined by future studies.

## 4. Materials and Methods

### 4.1. Ethical Statement and Pathological and Clinical Data

This study includes normal human and tumor tissue samples from the TIROCHUS collection, registered with the National Biobank Register of the National Institute of Health Carlos III (ISCIII, nº c.0003960) and from the Biobank of the Clinical University Hospital of Santiago de Compostela (CHUS). The use of these samples is in strict compliance with legislated procedures of personal and pathology data protection and has been approved by the regional Territorial Ethical Committee (CAEI 2015/238 and 2016/239). Pathological and clinical data from tissue samples include age, gender, and tumor type (including HercepTest^TM^ for breast cancer samples and Gleason score for prostate cancer samples).

### 4.2. Tissue Microarrays (TMA)

TMA were constructed following the standard technique previously described [66]. With the help of the H&E staining performed in every block of formalin-fixed paraffin-embedded (FFPE) tissue, two representative areas were selected for each condition (normal or cancer) to be included in the TMA. Core tissue diameter was 2 or 1.5-mm. TMA were made using a manual tissue arrayer MTA-1 (Beecher Instruments, Sun Prairie, WI, USA).

Initially for standardization purposes two TMAs were generated with cases of normal (non-neoplastic) thyroid, normal (non-neoplastic) breast, and prostate cancer, TMA-0a—three per group of each, and TMA-0b—three breast, and prostate cancer cases including mixed normal tissue in the thick cores. In those two TMAs, techniques to reduce background noise were tested and parallel immunohistochemistry (IHC) with DAB and TSA as substrates were performed as single staining for each receptor to compare results.

Next, two TMA were generated from FFPE tissue blocks with breast and prostate carcinoma including five cases of each group (Tables S1 and S2). All cases were blindly selected with an automated search engine in our database except for breast cancer where the search included marker evaluation (ERa, PR, HER2). Prostate cancer cases included different Gleason scores. For each case, paired normal tissue and cancer tissue from breast (MB, BC) or prostate (NP, PC) were included together with normal thyroid tissue (NT, patients older than 70 years old) as negative control. TMA-I included cases BC1/NB1, BC2, PC1/NP1 and PC2/NP2. TMA-II included cases BC3/MN3, BC4/MN4, BC5/MN5, PC3/NP3, PC4/NP4 and BC5/NP5 and NT1, NT2, and NT3.

A third TMA, TMA-III, was generated with another four triple negative breast cancer cases, two cores from each case, and one prostate cancer as control (Table S1 BC6-BC7-BC8 and BC9, and Figure S4). Of those, one was a medullary breast cancer and another was a lymph node metastasis.

Globally, we have tested *ColNu mIHCF* in cases of seven normal breast, eight normal prostates and six normal thyroid tissues, and 15 breast and 12 prostate cancers cases.

### 4.3. Prostate Needle-Core Biopsy

Needle-core biopsy sections obtained from transrectal biopsies (Table S3) were stained for standard ERa IHQ.

### 4.4. Immunohistochemistry (IHC)

3–4 µm sections of prostate needle-core biopsy were pretreated for antigen retrieval using PT Link with high pH (Dako-Agilent, Glostrup, Denmark). When using clinical conditions sections were stained using a standard automated staining (~30 min incubation with primary antibody, AutostainerLink 48 Stainer (Dako-Agilent) with EnVision^TM^ FLEX/HRP system (Dako-Agilent) for signal detection with DAB. When using *ColNu mIHC* conditions, all reagents were maintained but incubation with primary antibody was extended to overnight.

Diagnosis evaluation of hormone receptors was performed considering only positive nuclear staining, following CAP and ASCO Guidelines [23,24] and manufacturer’s instructions of ER/PR pharmDX kit (DAKO-Agilent) based in Allred scoring [67,68,69]. HER2 expression in breast cancer samples was evaluated using HercepTest^TM^ (Dako-Agilent). Gleason score was assigned according to the WHO criteria outlined in 2016 [22].

Since *ColNu mIHCF* was performed manually, IHC for each antibody was also repeated manually since only the last step, the substrate for the HRP, varies: DAB for IHQ and TSA for IHQF.

Antibodies and dilutions are shown in Table S4.

### 4.5. ColNu mIHCF

(See Supplementary *ColNu mIHCF* protocol for a step-by-step description and Table S4 for detailed confocal conditions).

Paraffin sections were pretreated as above using similar antigen retrieval in the PT Link system. Cyclic immunohistochemistry stainings were performed in the same sections/slides by using IVD validated antibodies used for diagnostic IHC (see Table S4). We used different Tyramide signal amplification (TSA, Perkin-Elmer, Waltham, MA, USA) as substrates for the peroxidase enzyme (HRP) linked to the secondary antibody (see Table S4). HRP links covalently TSA molecules to tyrosine amino acids of proteins [70,71]. Covalent bond resists heat and pH change while ionic bonding, characteristic of antigen-antibody binding, does not resist heat or pH out of physiological range (around 7–8). After each staining cycle, a denaturalization step induced by heat and pH, releases antibodies but maintains TSA signal in the section. Technical negative controls were run in parallel omitting primary antibodies by using only EnVision^®^ FLEX Antibody Diluent (Dako-Agilent, DM830).

At the end of the IHCF cycles, slides were incubated in 0.1% Sudan Black-B (Sigma, Steinheim, Germany) diluted in 70% ethanol for 20 min, washed three times with PBS-T (PBS with 0,02% Tween20) and mounted with Fluoro-Gel with Tris buffer (Electron Microscopy Sciences, Hatfield, PA, USA) including 2 mg/mL DAPI (D9542 Sigma).

Slides were photographed using a confocal microscope TC-SP5-AOBS with a white laser (470–670 nm) and a UV laser (Leica, Nussloch, Germany) equipped with the Leica Application Suite Advanced Fluorescence, LAS AF software, using serial sections (Z) every 1 µm for the 10× objective (PL APO 10×/0.40 CS) and 20× (PL APO 20×/N.A.0.70 CS) objective and 0.3 µm for the 63× objective (oil PL APO 63×/N.A.1.4-0.6 CS). Data were collected using Sequential Mode with the following order (Table S4): first, Channel 00 (DAPI) and Channel 01 (TSA-Cy5) together with transillumination for differential interference contrast (DIC Nomarski) per plane; second, Channel 02 (TSA-Fluorescein); third, Channel 03 (TSA-Cy3).

Data were collected at resolution 1024 × 1024 pixels, with zoom 1×–3×, giving an XY field of 912.7 × 912.7 µm for objective 10×, of a range from 775 × 775 µm till 288.5 × 288.5 µm for objective 20× and of a range of 246 × 246 µm till 90.4 × 90.4 µm for objective 63×. Thus the final resolution was between 0.89 µm/pixel (10×), 0.75–0.28 µm/pixel (20×) and 0.24–0.08 µm/pixel (63×). Each section on the TMA was systematically registered in at least 5 ROI at 20×, 10 pictures per ROI at 63×.

### 4.6. Quantification of the Cell Populations

Fiji (Image J) v1.5.1 (https://imagej.net/Fiji) [72] was used for automated counting with the a systematic procedure using the following tools: *Adjust Brightness/Contrast* to slightly adjust background for the automatic reconnaissance of nuclei; *Merge Channel* for identification of nuclei combining each channel, or more than one, with Ch00 (DAPI) allowing identification of single, double or triple positive nuclei; *Cell Counter* for manually counting each group of single, double or triple positive nuclei; *FreeHand* to select ROI when separating stroma from epithelium; *Clear Outside* to eliminate sections when counting a specific part of a picture.

Cell Profiler v3.0.0 (©Broad Institute, Harvard; https://cellprofiler.org) [73] was used for automated counting. Different modules must be organized into a Pipeline used for identification of nuclei. Our pipeline for counting 63× is describe step-by-step in Supplementary Methods. Of notice, at Steps 8–11 diameters must be adjusted for counting epithelial or stromal cells.

### 4.7. Graphs and Descriptive Statistics

Results are presented as Mean ± SEM using GraphPad Prism v.7 (GraphPad Software, San Diego, CA, USA).

## 5. Conclusions

In summary, our study is a proof of concept that *ColNu mIHCF* is a valid technique useful for cancer pathology. Our results suggest an immediate application in prostate cancer either to ameliorate diagnostic in needle-core biopsies or to propose additional therapies based on the expression of membrane ERa. With the advent of the new agonist and antagonist hormone therapies, we also foresee a future use of *ColNu mIHC* in the sub-classification of breast cancer types to adapt each case to a particular line of therapy, and in studies of tissue physiology of breast and prostate.

## Supplementary Materials

The following are available online at https://www.mdpi.com/2072-6694/12/12/3591/s1, Figure S1: Standardization of the IHCF using human thyroid sections; Figure S2: Quantification of ColNu mIHCF in the epithelial (left and centre) and stromal compartments (right); Figure S3: Signal from each individual channel obtained through ColNu mIHCF from breast cancer samples using confocal microscopy; Figure S4: ColNu mIHCF in and array with four additional triple negative breast cancer cases; Figure S5: Signal from each individual channel obtained through ColNu mIHCF from prostate cancer samples using confocal microscopy; Table S1: Paired normal breast and breast cancer cases used in TMA; Table S2: Paired normal prostate and prostate cancer cases used in TMA; Table S3: Needle-core biopsy samples; Table S4: Antibodies used in this work, including those clinically validated for In Vitro Diagnostic (IVD). Fluorophores, channels and conditions for sequentially registering at the confocal. Extended Methods: detailed ColNu mIHC protocol.

## Abbreviations

TSA	Tyramide signal amplification
TMA	Tissue microarray
IVD	In vitro diagnostics
ERa	Estrogen receptor alpha
PR	Progesterone receptor
AR	Androgen receptor
NB	Normal breast
BC	Breast cancer
NP	Normal prostate
PC	Prostate cancer
DIC	Differential interfering contrast
DAPI	4′,6-Diamidino-2-phenylindole
FFPE	Formalin-fixed paraffin-embedded
