# Supplementary material for: Sequential Colocalization of ERa, PR, and AR Hormone Receptors Using Confocal Microscopy Enables New Insights into Normal Breast and Prostate Tissue and Cancers
Source: Cancers (Basel). 2020 Nov 30;12(12):3591. doi: 10.3390/cancers12123591 (PMC7761237; doi:10.3390/cancers12123591)
Supplement: Supplementary file 1 [file cancers-12-03591-s001.pdf]

Supplementary Information

Supplementary Figures 1-2-3-4-5-6 page 2-15

Supplementary Tables 1-2-3-4 page 17-21

Supplementary Method: Detailed ColNu mIHC protocol page 22

Suppl. Figure 1

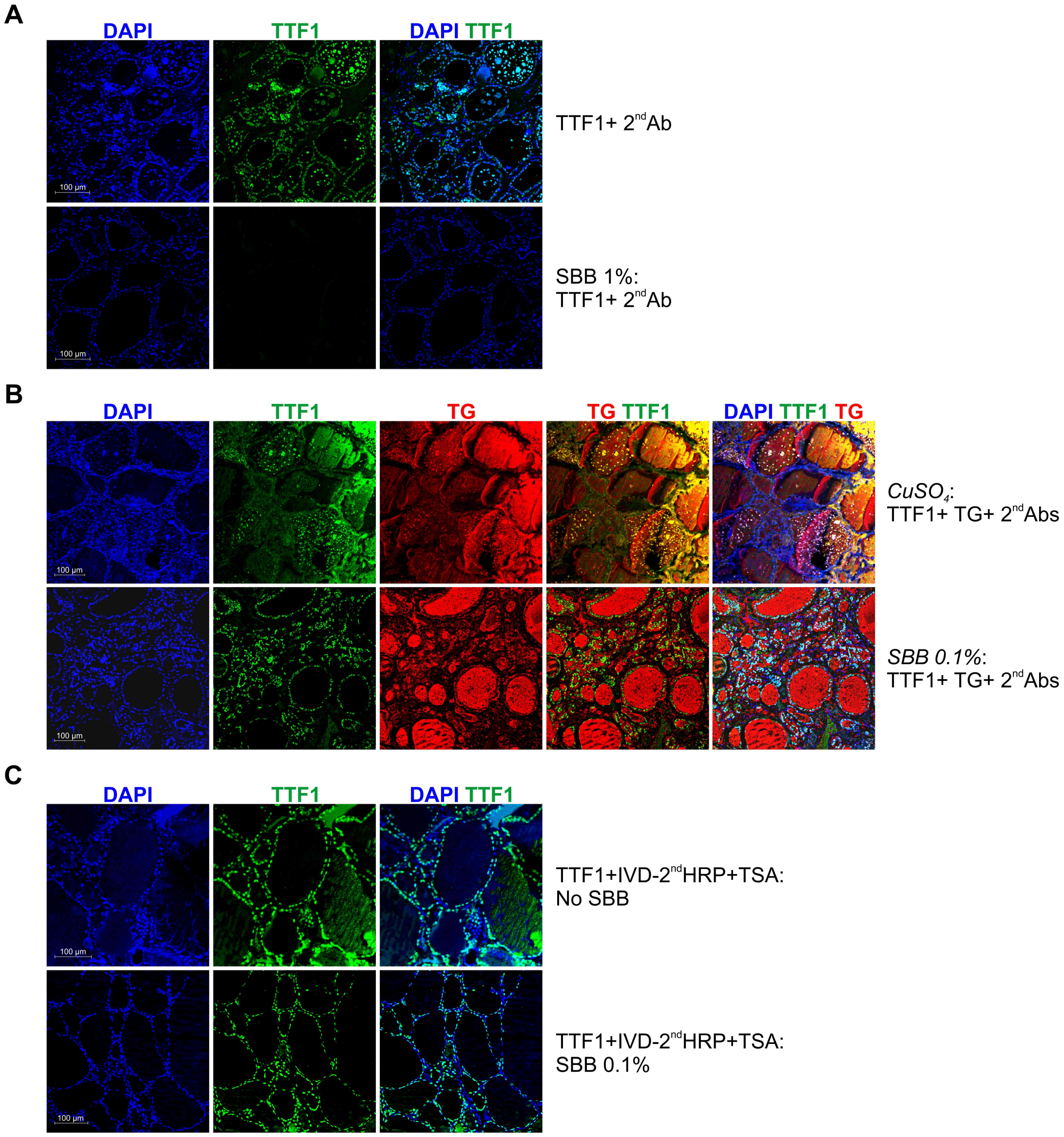

Supplementary Figure 1. Standardization of the IHCF using human thyroid sections. **A-B**
Immunofluorescence against the transcription factor TTF1 (IVD quality) or TG (IVD quality) with anti-mouse-Alexa 488 or anti-rabbit-Alexa 552 as secondary antibody (2_{nd} Ab), respectively. **A) Top:** Standard procedure with no quenching reveals nuclei stained for TTF1 and autofluorescence from follicular colloid (intense drops and green patches). Bottom: Pre-incubation with 1% SBB blocks TTF1 staining. **B)** Top: Double immunofluorescence for TTF1 and TG displaying staining and autofluorescence in both wavelength channels. Bottom: Incubation with 0.1% SBB after immunofluorescence and prior to mounting reduces autofluorescence, revealing very specific nuclear staining for TTF1 and cytoplasms with empty nuclei for TG that also accumulates in the colloid. **C)** Immunofluorescence anti-TTF1 using HRP-bound 2_{nd} Ab (IVD quality)
and TSA-Fluorescein without quenching (Top) shows strong nuclear signal together with autofluorescence. However, quenching with 0.1% SBB prior to mounting reveals an intense signal without autofluorescence. DAPI was added with the mounting medium to reveal the nuclei.

We performed high signal immunofluorescence, such as TTF1 or thyroglobulin (TG). TTF1 is a transcription factor abundantly expressed in the nucleus of epithelial follicular cells secreting cytoplasmic TG into the colloid. To err on the side of safety in the initial standardization, we used pre-adsorbed Alexa fluorescent-labelled secondary antibodies (fragment), not validated for IVD but with which we had extensive previous experience in FFPE sections from glucosaline-perfused (no blood) rats and human primary thyroid cultures. Routine immunofluorescence and mounting with DAPI for nuclear counter-staining was conducted followed by confocal microscopy; TTF1 was detected in the nuclei of the follicular epithelium as expected, but colloid autofluorescence was present, manifesting as intense drops or as a continuum inside the follicles (Suppl. Fig 1A, top). Pre-incubation with 1% SBB completely blocked the signal (Suppl. Fig 1A, bottom). For this reason, we compared routine staining to two other quenching methods performed *after* immunofluorescence. Incubation with 50 mM CuSO4 maintained the intense autofluorescence both in the λ492nm emission collected in the TTF1 immunofluorescence and the λ552nm emission collected in the Tg immunofluorescence (Suppl. Fig 1B, top). In contrast, incubation with 0.1% SBB resulted in a uniform, clear, and specific signal for both TTF1 (nuclear) and TG (cytoplasms with empty nuclei in epithelium, and colloid), significantly reducing autofluorescence (Suppl. Fig 1B, bottom).

Next, we applied this quenching procedure in TTF1 immunofluorescence substituting the fluorescent-labelled secondary antibody for the IVD *HRP-bound* secondary antibody routinely used for IHC in clinical pathology, using TSA-Fluorescein as substrate for the enzyme (Suppl. Fig 1C). This IVD secondary antibody is a mix of anti-rabbit and anti-mouse antibodies bound by a polymer to the enzyme horseradish peroxidase (HRP). Hereafter, we will refer to this procedure as IHCF. Using the usual clinical protocol, no quenching with SBB, nuclear signal for TTF1 in the follicular epithelium together with intense background colloid signal was obtained (Suppl. Fig 1C, top). When the same protocol was finished by an incubation with 0.1% SBB before mounting, the TTF1 signal was intense and specific without autofluorescence from the colloid (Suppl. Fig1C, bottom).

Suppl. Figure 2 A-B

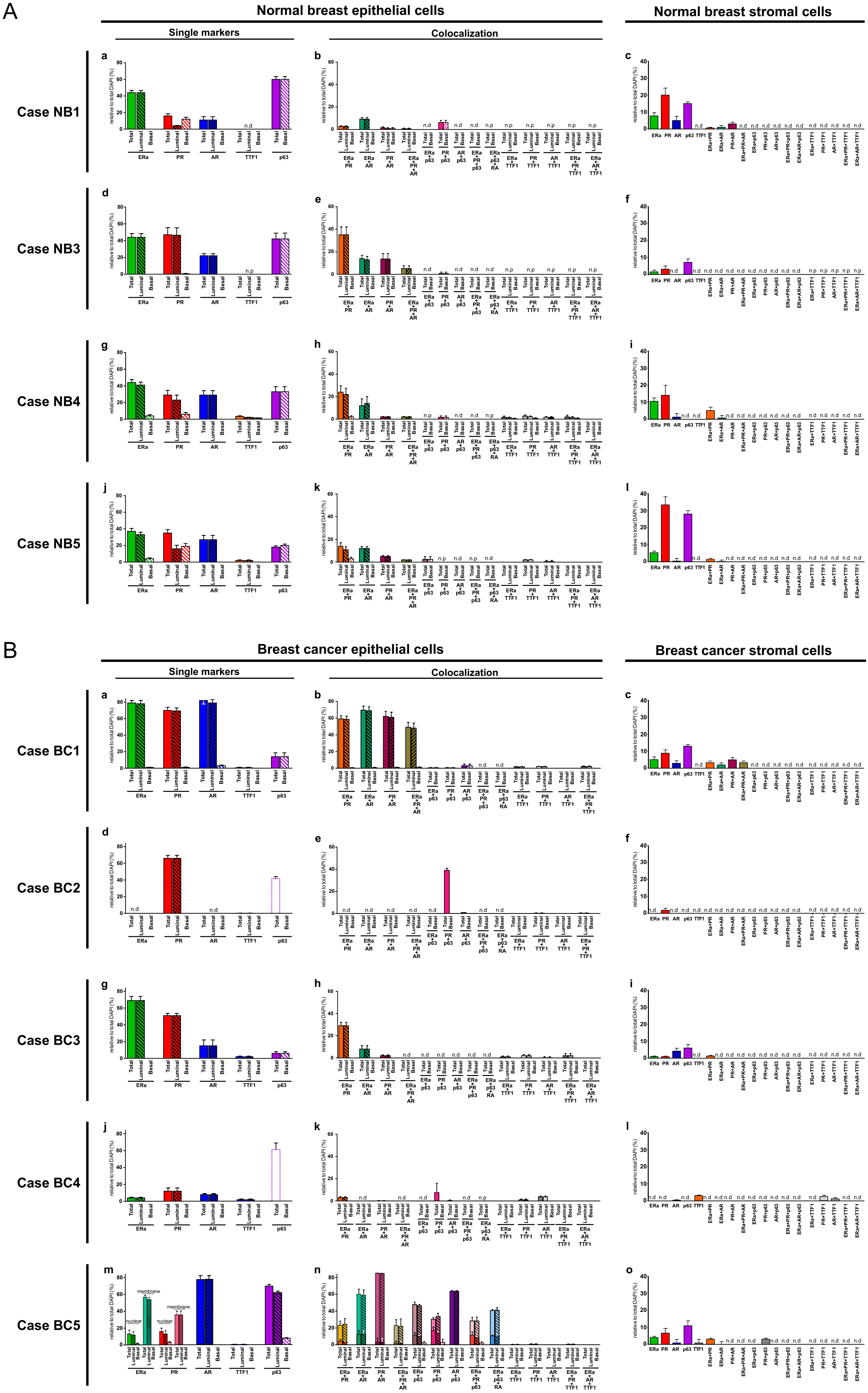

Suppl. Figure 2 C-D

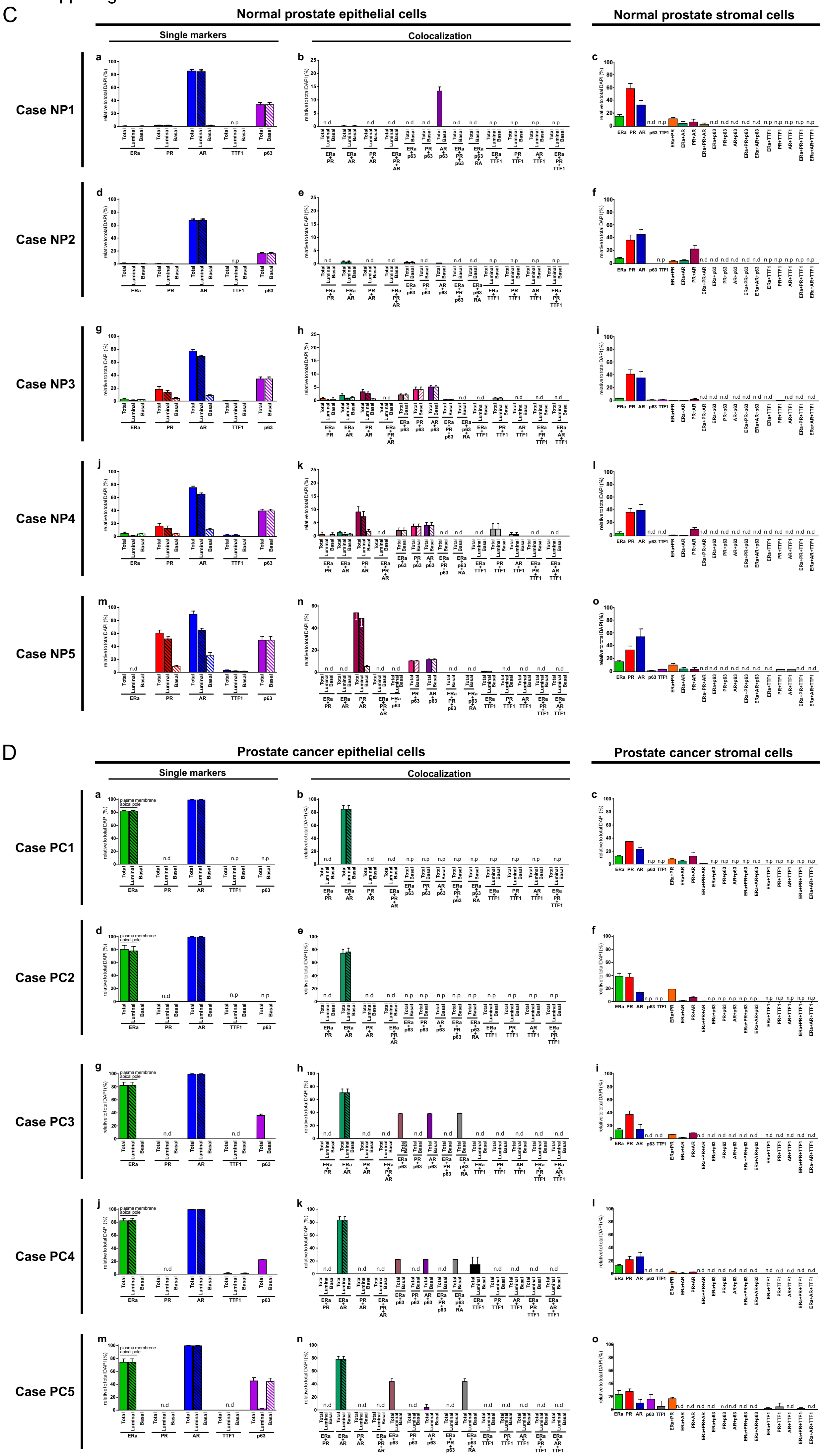

Supplementary Figure 2: Quantification of ColNu mIHCFs in the epithelial (left and centre) and stromal compartments (right).
Sections were segmented and single, double, or triple labelled cells were counted with automated platforms in repeated ColNu mIHCF stainings.
A) Normal breast counterpart: a–b-c) case NB1; **d-e-f)** case NB3; **g-h-i)** case NB4; **j-k-l)** case NB5.
B) Breast cancer: **a–b-c)** case BC1; **d-e-f)** case BC2; **g-h-i)** case BC3; **j-k-l)** case BC4; **m-n-o)** case BC5, in n soft colours indicate colocalization with plasma membrane ERa, and stronger colours show colocalization with nuclear ERa.
C) Normal prostate counterpart: a–b-c) case NP1; **d-e-f)** case NP2; **g-h-i)** case NP3; **j-k-l)** case NP4; **m-n-o)** case NP5.
D) Prostate cancer: a–b-c) case PC1; **d-e-f)** case PC2; **g-h-i)** case PC3; **j-k-l)** case PC4; **m-n-o)** case PC5.

Suppl. Figure 3 part I

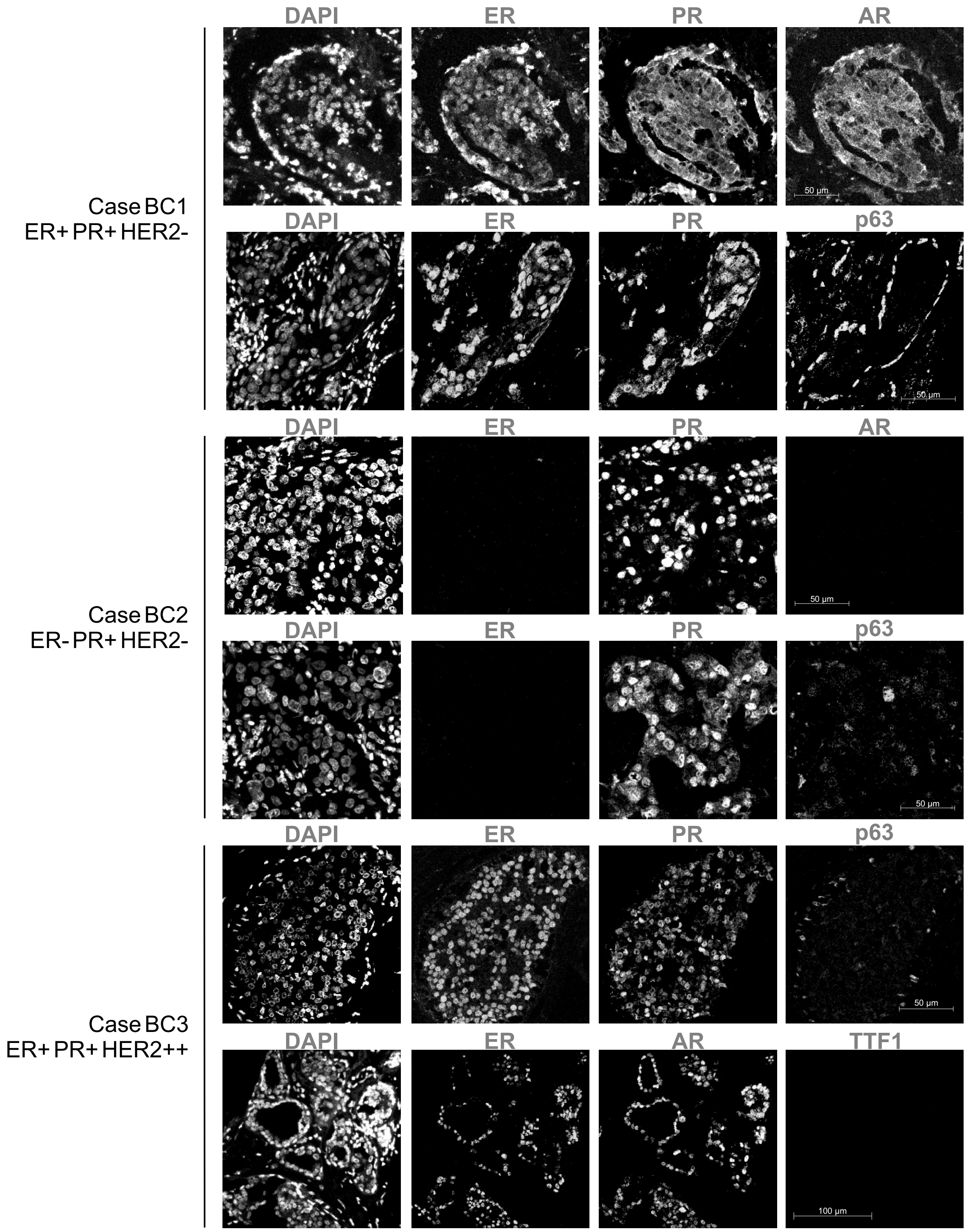

Suppl. Figure 3 part II

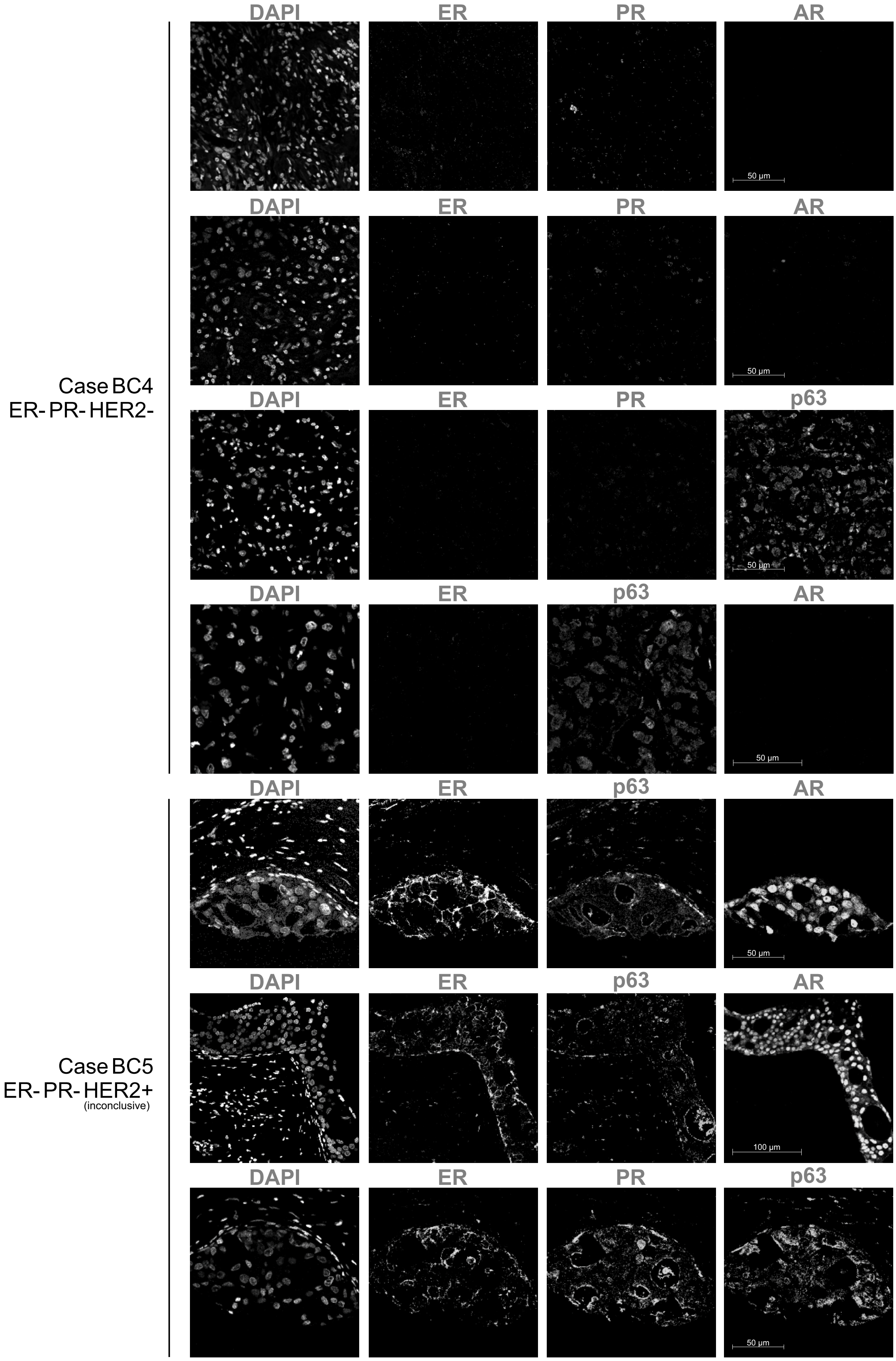

Supplementary Figure 3. Signal from each individual channel obtained through ColNu mIHCF from breast cancer samples using confocal microscopy.
The overlays are shown in figures 3 and 4.

Suppl. Figure 4 A-B

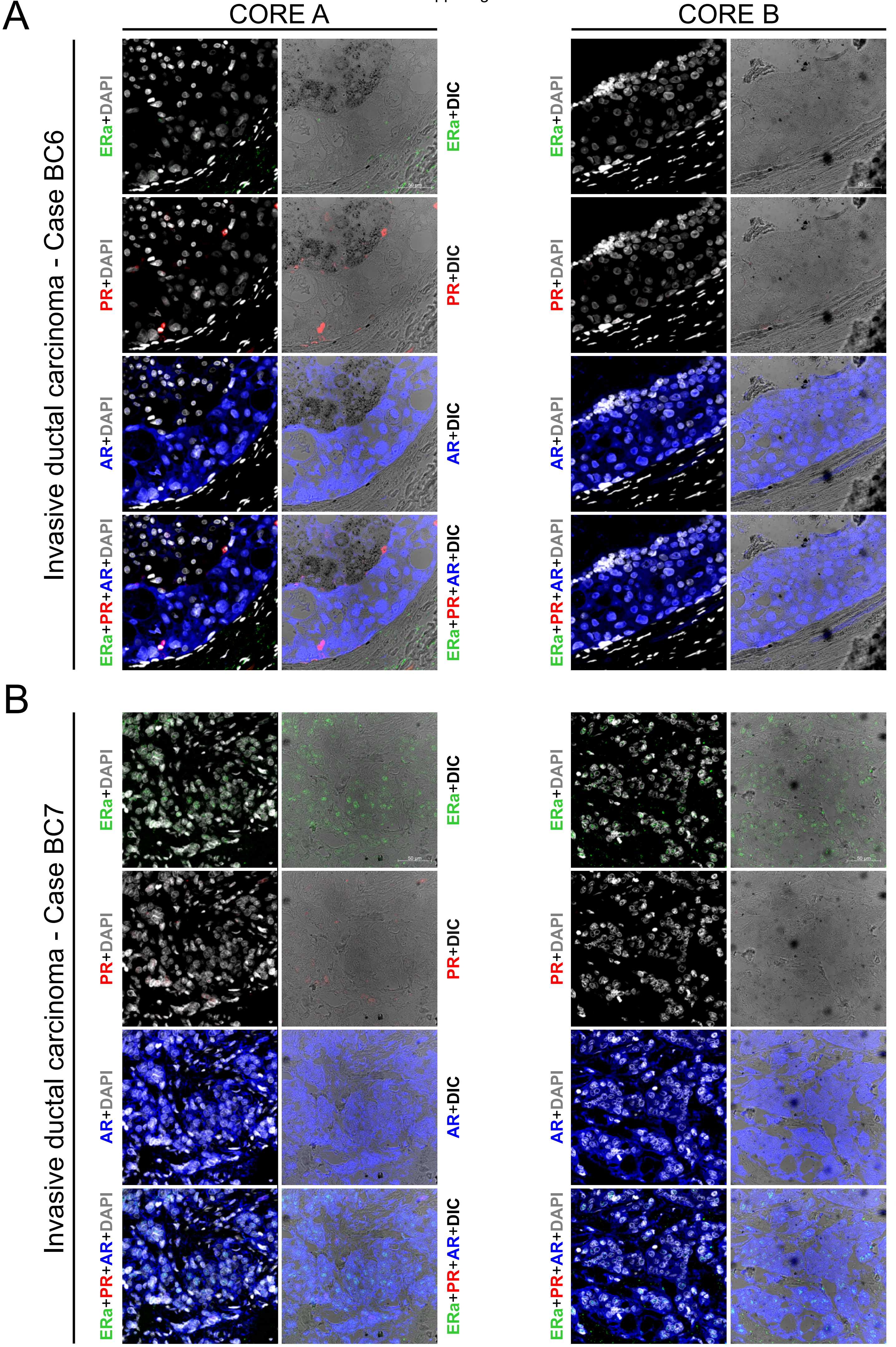

Suppl. Figure 4 C-D

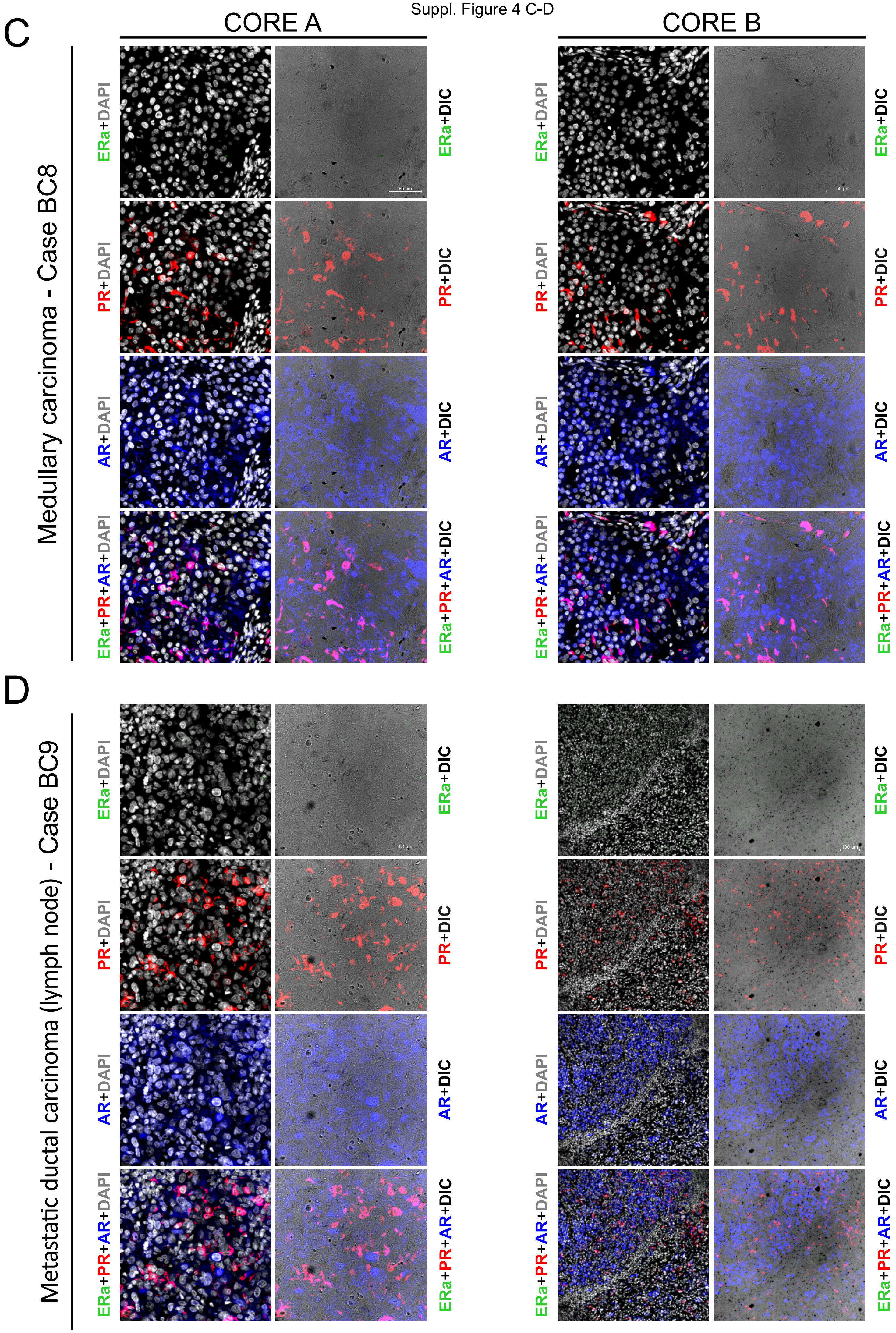

Supplementary Figure 4. ColNu mIHCF in and array with four additional triple negative breast cancer cases. Two thick tissue cores per case were included. Pathological and clinical data are summarized in Suppl. Table 1. Scale is indicated at the DIC channel. **A)** Case BC6, an invasive ductal carcinoma expressing AR but negative for ERa. PR was expressed in sporadic stromal cells. **B)** Case BC7, an invasive ductal carcinoma expressing AR and weak nuclear ERa. PR was expressed in sporadic stromal cells. **C)** Case BC8, a medullary breast carcinoma expressing AR but negative for ERa. Pleomorphic cancer cells with dendritic-like appearance expressed intense PR exclusively at the cytoplasm, colocalized with AR. **D)** Case BC9, a lymph node metastasis of a ductal breast carcinoma positive for AR but negative for ERa. Pleomorphic cancer cells with dendritic-like appearance expressed intense PR exclusively at the cytoplasm, colocalized with AR. Notice that field shown for Core B was performed with the Objective 20x while the others were with 63x.

DAPI was included in the mounting medium and reveals the nuclei. DIC: Differential Interfering Contrast with Nomarski and transillumination.

Suppl. Figure 5 part I

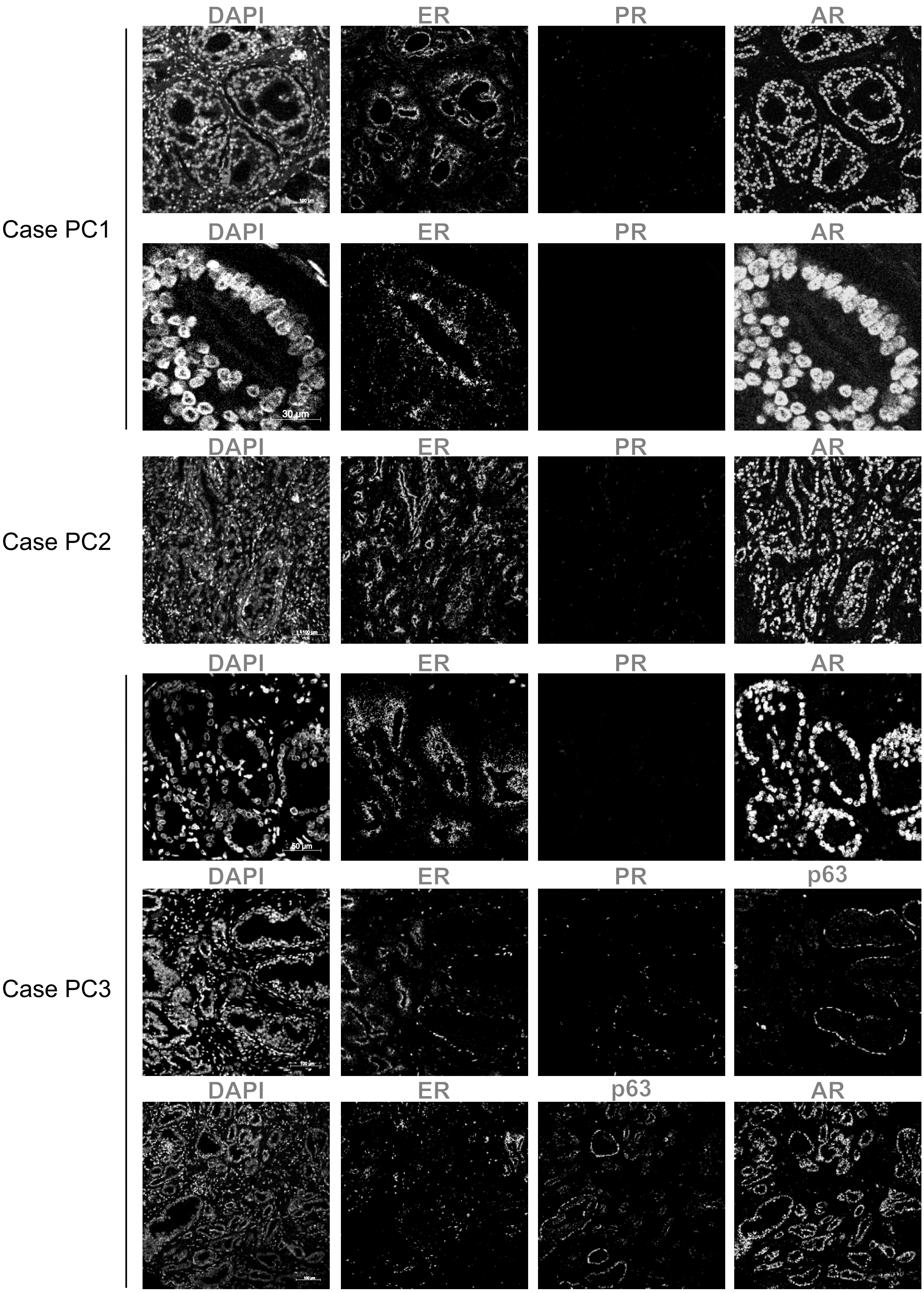

Suppl. Figure 5 part II

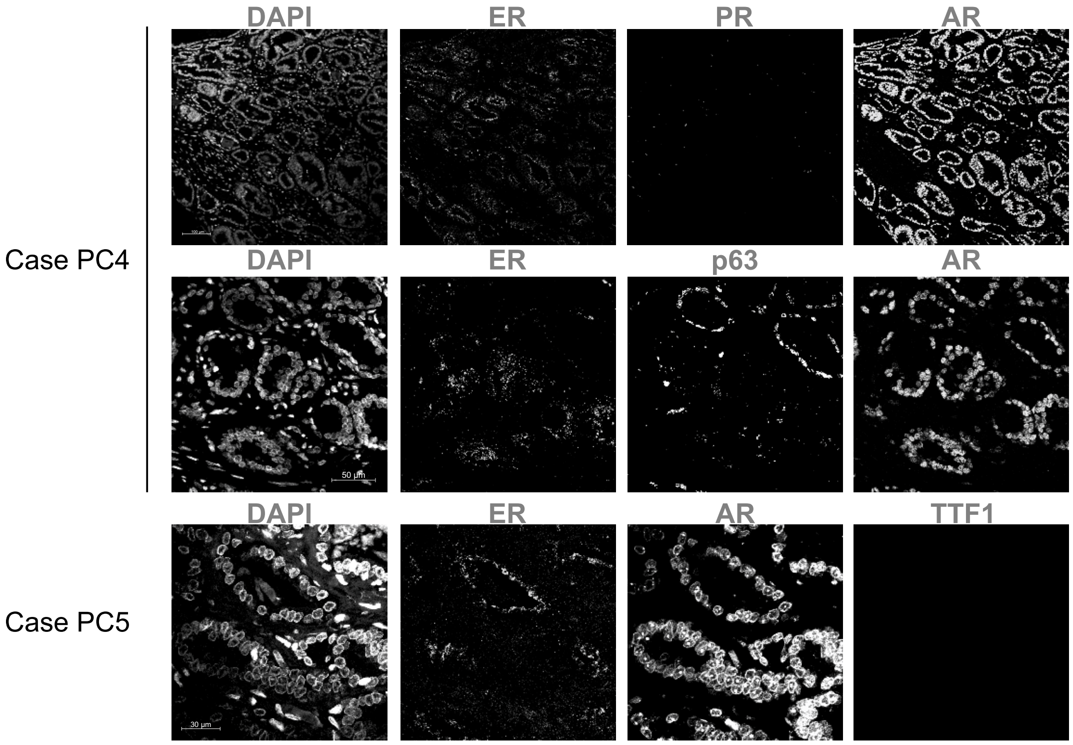

Supplementary Figure 5. Signal from each individual channel obtained through ColNu mIHCF from prostate cancer samples using confocal microscopy.
The overlays are shown in figure 6.

Table 1: Paired normal breast and breast cancer cases used in TMA

Case/Sample	Age	Gender	Diagnosis	Ki67 index	IHC* hormone receptor status (Allred score) HercepTest™
**TMA-I**					
► Case 1					
Normal tissue NB1 mixed up with BC1	77	Female	Invasive ductal carcinoma ER and PR+/HER2-	-	ERa: +/- weak in luminal cells (2+1=3)<br>PR: +/- weak in basal cells (1+1=2)<br>AR: + (2+1=3)
Breast cancer BC1				40%	ERa: + (5+3=8)<br>PR: + (5+3=8)<br>AR: + (4+2=6)<br>HER2: -
► Case 2					
Breast cancer BC2	56	Female	Invasive ductal carcinoma PR+ / ERa and HER2 -	37%	ERa: - (0)<br>PR: + (4+2=6)<br>AR: - (0)<br>HER2: -
**TMA-II**					
► Case 3					
Normal breast NB3	56	Female	Invasive ductal carcinoma Grade 2-3 Triple Positive	-	ERa: + (2+2=4)<br>PR: + (2+1=3)<br>AR: t.d.#
Breast cancer BC3				15%	ERa: + (4+2=6)<br>PR: + (3+1=4)<br>AR: t.d.#<br>HER2: ++
► Case 4					
Normal breast NB4	74	Female	Invasive ductal carcinoma Grade 3 Triple Negative	-	ERa: + (3+2=5)<br>PR: - (0)<br>AR: - (0)
Breast cancer BC4				71%	ERa: - (0)<br>PR: - (0)<br>AR: - (0)<br>HER2: -
► Case 5					
Normal breast NB5	58	Female	Invasive ductal carcinoma Grade 3 Triple negative	-	ERa: + (2+2=4)<br>PR: - (0)<br>AR: + (4+2=6)
Breast cancer BC5				23%	ERa: - (0)<br>PR: - (0)<br>AR: + (5+2=7)<br>HER2: + (inconclusive)
**TMA-III**					
BC6 (Core A&B)	46	Female	Invasive ductal carcinoma Grade 2 Triple negative	37%	ERa: - PR: - AR:+<br>HER2: + (inconclusive)
BC7 (Core A&B)	45	Female	Invasive ductal carcinoma Grade 3 Triple negative	67%	ERa: + PR: - AR:+<br>HER2: -
BC8 (Core A&B)	74	Female	Medular carcinoma Grade 3 Triple negative	51%	ERa: - PR: - AR:+<br>HER2: -
BC9 (Core A&B)	51	Female	Metastatic ductal carcinoma (lymph nodes) Primary tumor Grade 3 Triple negative	63%	ERa: - PR: - AR:+<br>HER2: + (inconclusive)

* *Performed in our TMAs with routine clinical conditions before ColNu mIHC*

\# *Technically deficient*

Table 2: Paired normal prostate and prostate cancer cases used in TMA

Case/Sample	Age	Gender	Diagnosis	Gleason score	IHC* hormone receptor status performed with ColNuc-mIHF conditions
**TMA-I**					
► Case 1					
Normal tissue NP1 mixed up with PC1	52	Male	Prostate adenocarcinoma	-	ERa: < 1%<br>PR: -<br>AR: +
Prostate cancer PC1				6 (3+3)	apical membrane ERa +<br>PR: -<br>AR: +
► Case 2					
Normal tissue NP2 mixed up with PC2	67	Male	Prostate adenocarcinoma	-	ERa: < 1%<br>PR: -<br>AR: +
Prostate cancer PC2				7 (4+3)	apical membrane ERa +<br>PR: -<br>AR: +
**TMA-II**					
► Case 3					
Normal prostate NP3	63	Male	Prostate adenocarcinoma	-	ERa: < 1%<br>PR: -<br>AR: +
Prostate cancer PC3				6 (3+3)	apical membrane ERa +<br>PR: -<br>AR: +
► Case 4					
Normal prostate NP4	67	Male	Prostate adenocarcinoma	-	ERa: 2-3%<br>PR: -<br>AR: +
Prostate cancer PC4				6 (3+3)	apical membrane ERa +<br>PR: -<br>AR: +
► Case 5					
Normal prostate with areas of prostatic hyperplasia NP5	52	Male	Prostate adenocarcinoma	-	ERa: < 1%<br>PR: + (whole cell)<br>AR: +
Prostate cancer PC5				9 (5+4)	apical membrane ERa +<br>PR: -<br>AR: +

* *Refered exclusively to epithelium, after results obtained in confocal microscopy*

Table 3: Needle-core biopsy samples

Case	Age	Gender	Diagnosis	Gleason score	ERa IHC
1	70	Male	Prostate adenocarcinoma	6 (3+3)	ERa+ apical membrane in cancer epithelium
2	86	Male	Prostate adenocarcinoma	10 (5+5)	ERa+ apical membrane in cancer epithelium
3	73	Male	Prostate adenocarcinoma	8 (4+4)	ERa+ apical membrane in cancer epithelium
4	76	Male	Prostate adenocarcinoma	6 (3+3)	ERa+ apical membrane in cancer epithelium
5	85	Male	No malignancy	-	ERa: -
6	72	Male	No malignancy	-	ERa: -

Table 4. Antibodies used in this work, including those clinically validated for *In Vitro Diagnostic* (IVD). Fluorophores, channels and conditions for sequentially registering at the confocal.

Antibody	Gene/ Protein	Antibody origin	Reference	Brand	Epitope	Antigen retrieval (PT-Link)	Dilution	Incubation
**ERa**	ESR1 Estrogen Receptor 1	Rabbit Ig G	EP1/ IR084 IVD	Dako	Full length recombinant human ERa. Epitope has been restricted between aa. 37-42: RPLGEV (Badve et al, 2013)	pH: 9	RTU	AutostainerLink 48 Stainer or 30 minutes (manual clinical conditions) or Overnight room Tª (ColNu mIHCF)
**PR**	PGR Progesterone Receptor	Mouse IgG1,kappa	PgR 636 / IR068 IVD	Dako	Full length PR isoform A formalin fixed. Epitope has been restricted at the N-term región aa. 164-594, a shared sequence between both isoforms A and B of PR.	pH: 9	RTU	AutostainerLink 48 Stainer or 30 minutes (manual clinical conditions) or Overnight room Tª (ColNu mIHCF)
**AR**	Androgen Receptor	Mouse IgG1	AR 441/ M352 IVD	Dako	Synthetic peptide aa 299-315 of human AR.	pH: 9	RTU	AutostainerLink 48 Stainer or 30 minutes (manual clinical conditions) or Overnight room Tª (ColNu mIHCF)
**TTF1**	NKX2-1	Mouse IgG1	SPT24 /NCL-L-TTF1 IVD	Novocastra	Recombinant protein fragment, expressed in bacteria, from aa 1-123 of the N-term end of human TTF1 (NKX2-1)	pH: 9	1:1000	AutostainerLink 48 Stainer or 30 minutes (manual clinical conditions) or Overnight room Tª (ColNu mIHCF)
**p63**	TP63	Mouse IgG2a	DAKO-p63/ IR662 IVD	Dako	Synthetic peptide located at the core of the DNA Binding Domain (DBD), aa 199-315 of human p63	pH: 9	RTU	AutostainerLink 48 Stainer or 30 minutes (manual clinical conditions) or Overnight room Tª (ColNu mIHCF)
**TG**	Thyroglobulin	Rabbit Ig G	Dako-GA50961-2 IVD	Dako	Human thyroglobulin isolated from human	pH: 9	RTU	AutostainerLink 48 Stainer or

					thyroid glands.			30 minutes (manual clinical conditions)<br>or<br>Overnight room Tª (ColNu mIHCF)
**EnVision™ FLEX/HRP system**	Secondary Ab	Mix of goat anti-rabbit and goat anti-mouse antibodies covalently linked to a Dextran polymer containing HRP	IVD	Dako		-	RTU	AutostainerLink 48 Stainer<br>or<br>30 minutes (both for manual clinical conditions and ColNu mIHCF)
**Alexa 488 Donkey anti-mouse secondary antibody (no IVD)**	Secondary Ab used during standardization for TTF1 detection in human thyroid	Donkey	AB_2340850 Code: 715-546-151	Jackson Immuneresearch Europe	Alexa Fluor® 488 AffiniPure $F(ab')_2$ Fragment Donkey Anti-Mouse IgG (H+L) Minimal Cross Reactivity after pre-adsorbtion with Bovine, Chicken, Goat, Guinea Pig, Syrian Hamster, Horse, Human, Rabbit, Rat, Sheep Serum Proteins	-	1:1000	1 hour
**Cy3 Donkey anti-rabbit secondary antibody (no IVD)**	Secondary Ab used during standardization for Tg detection in human thyroid	Donkey	AB_2313568 Code: 711-166-152	Jackson Immuneresearch Europe	Cy™3 AffiniPure $F(ab')_2$ Fragment Donkey Anti-Rabbit IgG (H+L)	-	1:1000	1 hour
**TSA Fluorescein**			NEL760001KT	Perkin Elmer			1:200	5 minutes
**TSA Cyanine 3**			NEL760001KT o NEL744001KT	Perkin Elmer			1.200	5 minutes
**TSA Cyanine 5**			NEL760001KT	Perkin Elmer			1:100	10 minutes
**DAPI**			D9542	Sigma			2 mg/ml	Included in Mounting medium Fluoro-Gel with Tris buffer, (Electron Microscopy Sciences, Hatfield)

**Channel**	**Fluorophore**	**λEx (nm)**	**λEm (range; nm)**	**Pseudocolour**
Ch00<br>+<br>Transillumination<br>+	DAPI<br><br>DIC Nomarski	405	414 - 479	Grey<br><br>Phase contrast
Ch01	TSA Cyanine 5	640	670 - 770	Blue
Ch02	TSA Fluorescein	492	499 - 547	Green
Ch03	TSA Cyanine 3	552	565 – 624	Red

RTU, Ready-to-use; aa, amino acid; IVD, in vitro diagnostic

ColNu mIHC protocol

1st Cycle

1. Block with EnVision® FLEX Peroxidase-Blocking Reagent, (DAKO-Agilent) for 20 minutes. TSA is based on a reaction catalyzed by peroxidase (HRP) enzyme; it is essential to be sure that there is no endogenous HRP activity.
2. Wash in a Hellendhal glass coplin jar with TBS 1X+ 0.1% Tween20 (Sigma, Saint Louis) for 5 minutes and 2 times in PBS for 5 minutes each.
3. Block slides in a Hellendhal glass coplin jar with Tropix I-BLOCK (Thermo Fisher Scientific) (1:2 dilution in PBS) for 2 hours at room temperature (RT)
4. Wash in a Hellendhal glass coplin jar with TBS 1X+ 0.1% Tween20 (Sigma, Saint Louis) for 5 minutes and 2 times in PBS for 5 minutes each.
5. Incubate with primary antibody overnight at room T^a in a humidified chamber.
6. Wash in a Hellendhal glass coplin jar with TBS 1X+ 0.1% Tween20 for 5 minutes and 2 times in PBS for 5 minutes each.
7. Incubate with secondary antibody EnVision® FLEX/HRP for 30 minutes at RT.
8. Wash in a Hellendhal glass coplin jar with TBS 1X+ 0.1% Tween20 for 5 minutes and 2 times in PBS for 5 minutes each.
9. Incubate with TSA-Fluorescein (NEL760001KT, Perkin Elmer) 1:200 diluted in 1X Plus Amplification Diluent (Perkin Elmer) for 5 minutes at room T^a.
10. Wash in a Hellendhal glass coplin jar with TBS 1X+ 0.1% Tween20 for 5 minutes and 2 times in PBS for 5 minutes each.

2nd Cycle

11. Place four similar glass slides on the bottom of a plastic container (12×9×6 cm, the outer container of a 1 ml blue filter tips' box) filled with 200 mL of 10 mM TE buffer, pH 9.0. Your slide should replace one of the middle positions. Put the box carefully inside a 700-W household microwave oven. First heat the TE buffer at 100% power but prevent boiling (~2.25 minutes, standardize carefully with a timer), and then continue heating for another 15 minutes at 20% power.
12. Allow slides to cool in the TE buffer for 30 minutes at room T^a.
13. Wash in PBS for 3 minutes.
14. Repeat steps 1 to 10 using a different primary antibody (2nd cycle) but similar secondary antibody, and replacing the previous TSA for TSA-Cyanine 3 (NEL760001KT or NEL744001KT, Perkin Elmer) diluted 1:200 in 1X Plus Amplification Diluent for 5 minutes at room T^a.

3rd Cycle

15. Repeat steps 11 to 13 to wash out the second cycle of antibodies
16. Repeat steps 1 to 10 using a different primary antibody (3rd cycle) but similar secondary antibody, and replacing the previous TSA for TSA-Cyanine 5 (NEL760001KT, Perkin Elmer) diluted 1:100 in 1X Plus Amplification Diluent for 10 minutes at room T^a (notice that this fluorophore needs more concentration and time).

**Removing background**	17. Incubate the slides in a Hellendhal glass coplin jar with 0.1 % Sudan Black B (Sigma, Saint Louis) diluted in 70% ethanol, for 20 minutes at room T^a.<br>18. Wash 3 times in PBS+0.02% Tween20 for 5 minutes each.<br>19. Finally rinse each slide carefully with the same buffer to remove visible excess of Sudan Black B.
**Mounting**	20. Sections were mounted with a glass coverslip (0.17 µm) applying 1-2 drops of mounting medium (FluoroGel with Tris buffer, 17985-10, Electron Microscopy Sciences) containing 2 mg/mL DAPI (D9542, Sigma) for nuclei staining. Put the coverslip on top, avoiding bubbles.<br>21. , Allow to dry at room T^a for 5-10 minutes.<br>22. Store in the dark at 2-8º C (cold room) until visualization.
